# High-intensity interval training alleviates depressive-like behaviors and is associated with differences in gut microbiota composition and prefrontal LPS-associated inflammatory measures in CUMS rats

**DOI:** 10.3389/fcimb.2026.1929000

**Published:** 2026-10-01

**Authors:** Min-Xia Huang, Shi-Yu Xia, Hua-Zhe Wan, Xing-Jian Jiang, Qiong Wu, Zhigang Gong, Zhen Lyu

**Affiliations:** 1 Science and Technology College of NCHU, Jiujiang, China; 2 Key Lab of Aquatic Sports Training Monitoring and Intervention of General Administration of Sport of China, Faculty of Physical Education, Jiangxi Normal University, Nanchang, China; 3 School of Psychology, Shanghai University of Sport, Shanghai, China

**Keywords:** chronic unpredictable mild stress, depression, gut microbiota, high-intensity interval training, LPS-related inflammation

## Abstract

**Background:**

Exercise is a safe and effective intervention for improving depressive symptoms, but the effects and related mechanisms of high-intensity interval training (HIIT) remain unclear. Growing evidence suggests that interactions among the gut microbiota, immune-inflammatory responses, and the central nervous system may contribute to the development and progression of depression. This study investigated whether a 4-week HIIT intervention attenuated depressive-like behaviors and explored related differences in gut microbiota composition and prefrontal cortical lipopolysaccharide (LPS)-associated inflammatory measures.

**Methods:**

Forty male Sprague-Dawley rats were initially assigned to either the chronic unpredictable mild stress (CUMS) or control conditions. Following CUMS modeling and corresponding control procedures, rats were further allocated to four groups: CUMS_Sed, CUMS_Ex, Con_Sed, and Con_Ex. Exercise groups performed HIIT on motorized treadmills. Depressive-like behaviors were assessed using the sucrose preference test and the open field test, and body weight was monitored longitudinally as a secondary physiological outcome during CUMS exposure. Gut microbiota composition was profiled using 16S rRNA gene sequencing. LPS levels in the prefrontal cortex and ileum, and IL-1β levels in the prefrontal cortex, were measured using enzyme-linked immunosorbent assay, and prefrontal cortical Toll-like receptor 4 (*TLR4*) and *Rela* (NF-κB p65/RelA) mRNA expression levels were assessed by reverse transcription quantitative PCR.

**Results:**

CUMS induced marked depressive-like behaviors, including reduced sucrose preference and altered open field test performance. HIIT partially attenuated these behavioral alterations. In exploratory microbiota analyses, HIIT-treated CUMS rats differed from sedentary CUMS rats in gut microbial diversity and taxonomic composition and also showed lower prefrontal cortical LPS and IL-1β levels and lower *TLR4* and *Rela* mRNA expression. In contrast, IL-1β was higher in exercised control rats, and ileal LPS levels were lower overall in exercised rats.

**Conclusions:**

These findings suggest that HIIT-related improvements in depression-related phenotypes were accompanied by attenuation of LPS-associated inflammatory changes in the prefrontal cortex, while exploratory microbiota analyses identified differences in gut microbiota composition. HIIT may represent a promising exercise-based strategy for improving stress-related depressive-like phenotypes.

## 1 Introduction

Depression is a common and disabling psychiatric disorder characterized by high prevalence, a high risk of relapse, and a substantial socioeconomic burden (Greenberg et al., 2021; Desai Boström et al., 2025; Rong et al., 2025). Its core clinical features include persistent low mood and diminished interest, and it is often accompanied by varying degrees of cognitive impairment (Marx et al., 2023). The onset and progression of depression are jointly shaped by genetic susceptibility, environmental stressors, and multisystem biological processes (Cui et al., 2024; Meng et al., 2024; Feng et al., 2025). Accumulating evidence indicates that depression cannot be fully explained by localized abnormalities within the central nervous system alone, such as altered prefrontal cortical function, but may also involve disrupted coupling between the central nervous system and peripheral physiological systems (Ménard et al., 2017; Chan et al., 2023; Nusslock et al., 2024). Accordingly, elucidating the mechanisms that govern central-peripheral interactions in depression has become a major focus of contemporary research on its pathophysiology.

The gut-brain-immune axis is increasingly recognized as an important framework for understanding interactions between central and peripheral systems in depression (Zhu et al., 2025). This axis is commonly conceptualized as a bidirectional communication network encompassing the central nervous system, the gut microbial ecosystem, and the intestinal mucosal barrier, and it transmits signals through neural, immune-inflammatory, and metabolic pathways, including those related to lipopolysaccharide (LPS), Toll-like receptor 4 (TLR4), and nuclear factor kappa B (NF-κB) (Aburto and Cryan, 2024; He et al., 2024; O’Riordan et al., 2025). Emerging evidence further suggests that interventions for depression, such as exercise, may exert their benefits at least in part by modulating the gut-brain-immune axis, but the specific mechanisms remain incompletely understood (Donoso et al., 2023; Souza et al., 2023).

Exercise is a safe and health-promoting non-pharmacological intervention that has been shown to alleviate depressive symptoms (Noetel et al., 2024; Liu et al., 2025). Its antidepressant effects may be mediated through multiple pathways, including modulation of the gut microbial community, maintenance of intestinal mucosal barrier homeostasis, and regulation of immune and inflammatory responses (Park et al., 2025; Yao et al., 2025). Recent studies further suggest that, in addition to continuous training, high-intensity interval training (HIIT) may alleviate depression-related symptoms or phenotypes, and its effects may involve coordinated regulation across multiple physiological systems (Tao et al., 2024; Yao et al., 2025). However, despite growing interest in the potential benefits of HIIT, it remains insufficiently understood whether HIIT improves depression-related phenotypes and whether these effects are associated with differences in gut microbiota composition and LPS-associated inflammatory changes.

Accordingly, we used a chronic unpredictable mild stress (CUMS) rat model to systematically evaluate the effects of HIIT on depressive-like behaviors and to further explore whether these effects were associated with differences in gut microbiota composition and prefrontal cortical LPS-associated inflammatory changes. Depressive-like behaviors were assessed using the sucrose preference test and the open field test, gut microbiota composition was profiled by 16S rRNA sequencing, and inflammation-related markers were measured in the ileum and prefrontal cortex, including LPS levels in both tissues, as well as interleukin-1β (IL-1β) levels and *TLR4* and *Rela* (NF-κB p65/RelA) mRNA expression in the prefrontal cortex. We hypothesized that HIIT would ameliorate CUMS-induced depressive-like phenotypes and that these effects would be accompanied by differences in gut microbiota composition and attenuation of LPS-associated inflammatory measures, including lower prefrontal cortical *TLR4* and *Rela* mRNA expression. This study may provide experimental evidence regarding biological changes associated with the beneficial effects of HIIT on depression-related phenotypes and inform the future optimization of exercise interventions.

## 2 Materials and methods

### 2.1 Animals

Forty 6-week-old specific pathogen-free (SPF) male Sprague-Dawley rats weighing 180–220 g were obtained from Hunan SJA Laboratory Animal Co., Ltd. (Changsha, Hunan, China). Upon arrival, the rats were housed under a 12-h light/dark cycle with *ad libitum* access to water except during scheduled water-deprivation stressors. Throughout the experiment, all rats were maintained under the same baseline feeding regimen. Food was provided once daily at 18:00, with intake restricted to 15 g per rat per day, except when food deprivation was specifically scheduled as a CUMS stressor. Following completion of a scheduled food-deprivation stressor, the standard feeding regimen was resumed. All experimental procedures were conducted in accordance with the National Institutes of Health guidelines and were approved by the Experimental Animal Ethics and Welfare Committee of the College of Physical Education, Jiangxi Normal University (JXNU-PEC-2020003).

After 1 week of acclimation, rats were assigned to either the chronic unpredictable mild stress (CUMS) group or the control (Con) group (n = 20 per group). Group assignment was based on body weight using a randomized block design as previously described (Festing, 2014). After completion of the CUMS procedure, rats were further allocated to four groups (n = 10 per group) using the same design criteria: CUMS sedentary (CUMS_Sed) group, CUMS exercise (CUMS_Ex) group, control sedentary (Con_Sed) group, and control exercise (Con_Ex) group. One rat in the CUMS_Ex group died during week 3 of HIIT. Longitudinal body-weight and behavioral analyses and post-intervention biochemical analyses included nine CUMS_Ex rats and ten rats in each of the other groups. The week-2 Vmax reassessment was completed before this death, and Vmax analysis included ten rats in each exercise group. Body weight was measured weekly at the same time of day.

### 2.2 CUMS model

The CUMS protocol was adapted from previously published methods (Willner, 2017) with appropriate modifications. The paradigm consisted of two CUMS exposure phases separated by a 2-day behavioral-testing interval. After the first 2 weeks of CUMS exposure, no additional CUMS stressors were administered during the subsequent 2 days, during which behavioral testing was conducted. CUMS exposure was then resumed after completion of the behavioral assessments. During the first 10 CUMS exposure days, rats were exposed to one stressor per day, whereas during the remaining CUMS exposure days, rats were exposed to two stressors per day according to the predefined schedule. Stressors were arranged in a randomized order. Eight types of stressors were used, including food deprivation for 24 h, water deprivation for 24 h, cold water swimming at 4 °C for 5 min, light/dark cycle reversal for 24 h, noise exposure at 110 dB for 2 h, cage tilting at 45°for 24 h, tail pinch for 2 min, and wet bedding for 24 h. To maintain unpredictability, the same stressor was not applied on two consecutive CUMS exposure days, and the total number of applications for each stressor did not exceed six throughout the entire protocol. Stressors requiring 24-h exposure were initiated at 18:00 and terminated at 18:00 on the following day. Control rats underwent comparable handling procedures but were not exposed to chronic stressors (**Figure 1**).

**Figure 1:**
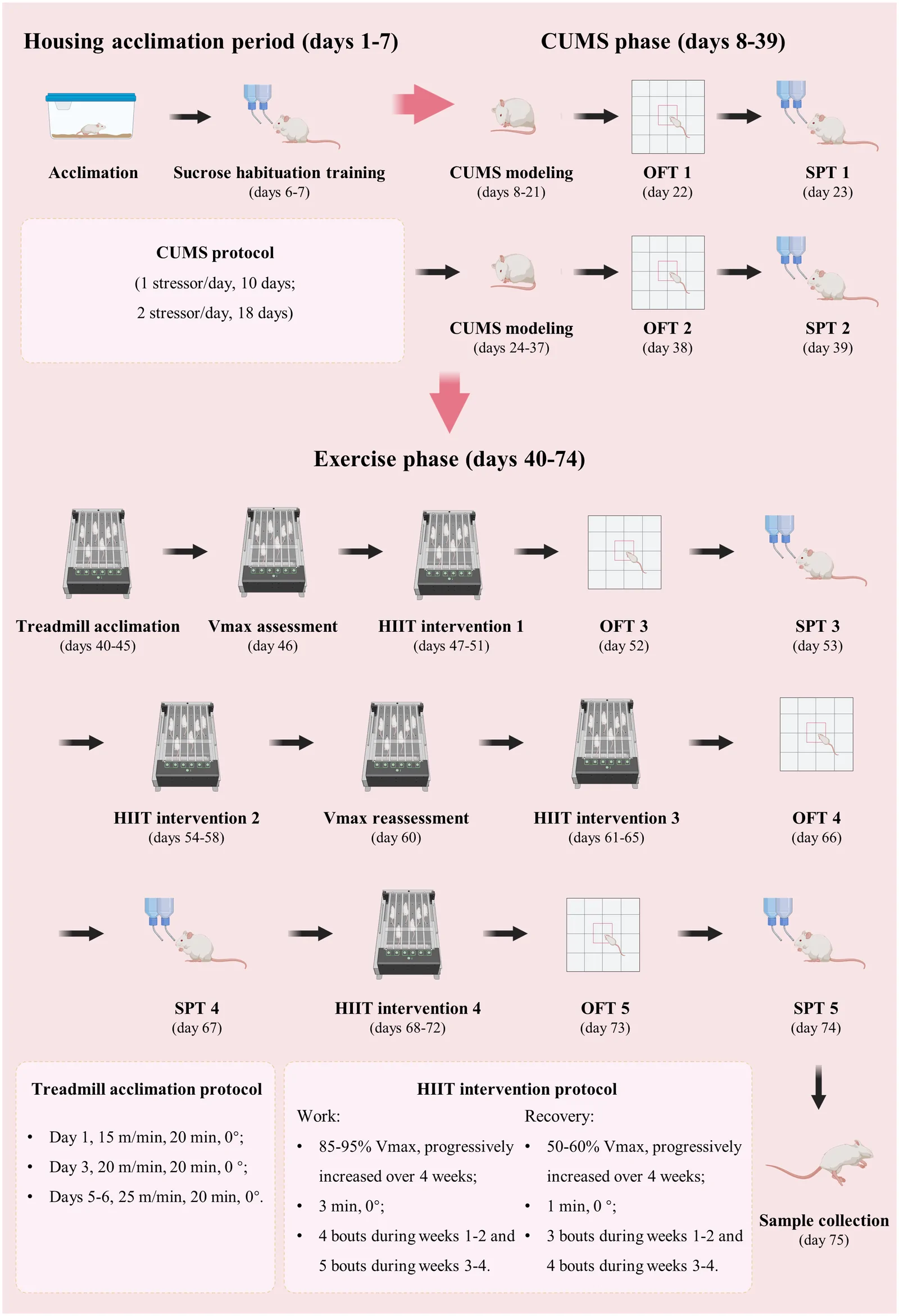
Experimental flowchart. SPT, sucrose preference test; OFT, open field test; CUMS, chronic unpredictable mild stress; HIIT, high-intensity interval training; Vmax, maximal running speed.

### 2.3 Exercise intervention

#### 2.3.1 Treadmill acclimation

After completion of the CUMS model, rats in the Con_Ex group and the CUMS_Ex group underwent treadmill acclimation on days 40–45 using motorized treadmills (Shanghai Xinxin Information Technology Co., Ltd., Shanghai, China). The acclimation period included four training days and two rest days. Rats ran at 15 m/min for 20 min on acclimation day 1, at 20 m/min for 20 min on day 3, and at 25 m/min for 20 min on days 5 and 6. Rats rested on days 2 and 4. The treadmill incline was maintained at 0° throughout the acclimation sessions. The detailed acclimation protocol is shown in **Table 1**.

**Table 1:** Treadmill acclimation protocol.

Study days	Day 40	Day 42	Days 44-45
Speed	15 m/min	20 m/min	25 m/min
Running duration	20 min	20 min	20 min
Incline	0°	0°	0°

Rats in the exercise groups completed four treadmill acclimation sessions over days 40–45 and rested on days 41 and 43. The listed running duration refers to the treadmill acclimation bout and excludes the 5-min warm-up and 5-min cool-down performed at 15 m/min.

#### 2.3.2 HIIT program

Following completion of treadmill acclimation on day 45, maximal running speed (Vmax) was assessed in the exercise groups on day 46 using an incremental workload test. Rats first completed a warm-up at 20 m/min for 5 min and then rested for 4 min. The treadmill speed was subsequently increased by 5 m/min every 3 min until exhaustion. Exhaustion was defined as the point at which a rat could no longer keep pace with the treadmill despite receiving a 10-s electrical stimulus (Kim et al., 2020). The Vmax obtained from this test was used to prescribe the training intensity as predetermined percentages of individual maximal running capacity for each rat. To account for training-induced changes in running capacity, Vmax was reassessed on day 60 after completion of the first 2 weeks of HIIT, and the training speeds for the remaining 2 weeks were recalculated using the updated individual Vmax values.

The HIIT protocol was adapted from previously published methods (Bittencourt et al., 2020). Training was conducted 5 days per week for 4 weeks, with HIIT sessions performed on days 47-51, 54-58, 61-65, and 68-72. Each session consisted of alternating 3-min high-intensity work bouts and 1-min low-intensity recovery bouts. The detailed protocol is shown in **Table 2**. Electrical stimulation was used only during the Vmax tests, whereas during routine HIIT sessions, gentle tactile prompting with a wooden stick was used when necessary to encourage the rats to maintain the prescribed running speed.

**Table 2:** HIIT training protocol.

Training parameter	Week 1	Week 2	Week 3	Week 4
Study days	47-51	54-58	61-65	68-72
Work intensity	85% Vmax	90% Vmax	90% Vmax	95% Vmax
Work bout duration	3 min	3 min	3 min	3 min
Number of work bouts	4	4	5	5
Recovery intensity	50% Vmax	55% Vmax	55% Vmax	60% Vmax
Recovery bout duration	1 min	1 min	1 min	1 min
Number of recovery bouts	3	3	4	4
Incline	0°	0°	0°	0°

Rats in the exercise groups performed HIIT on 5 days per week for 4 weeks. Each training session included a 5-min warm-up and a 5-min cool-down at 15 m/min. Recovery bouts were performed between consecutive high-intensity work bouts, resulting in one fewer recovery bout than work bout. Training intensities during weeks 1–2 were prescribed according to the Vmax measured on day 46, whereas intensities during weeks 3–4 were based on the reassessed Vmax on day 60. Vmax, maximal running speed.

During both the acclimation period and the HIIT intervention, rats in the exercise groups underwent a 5-min warm-up at 15 m/min before each training session and a 5-min cool-down at the same speed after each session. Sedentary rats were handled similarly and exposed to the treadmill environment without running to minimize handling-related differences among groups.

### 2.4 Behavioral testing

#### 2.4.1 Open field test

The open field test (OFT) was conducted according to previously published methods (Wang et al., 2009). The open field apparatus consisted of a black box measuring 1.0 m × 1.0 m × 0.7 m (length × width × height). The floor was divided into 25 equal squares. Before testing, rats were transferred to the testing room and allowed to acclimate for 30 min. At the start of the test, each rat was gently placed in the central area of the open field. The experimenter then exited the room, and the rat was allowed to explore freely for 5 min. All testing was conducted under dim lighting conditions. After the session, the rat was returned to its home cage.

Behavior in the apparatus was recorded using a video camera, including rearing frequency, number of grid crossings, time spent in the center zone (defined as the central nine squares), and time spent in the corner zone (defined as the four corner squares). The remaining 12 squares were not included in either zone-duration measure. Rearing was defined as each occurrence in which both forepaws were lifted off the floor, either unsupported or supported against the wall. A grid crossing was counted when at least three paws entered an adjacent square. Center- and corner-zone occupancy was determined using the same criterion, with zone duration recorded when at least three paws were within the center zone or one of the corner squares, respectively. To minimize potential odor cues, the apparatus was cleaned with 75% ethanol and allowed to air dry between tests. During the CUMS modeling period, the OFT was conducted after the first CUMS exposure phase and again following completion of the second CUMS exposure phase. Two OFT assessments were conducted during the HIIT intervention, followed by a final post-intervention assessment. Each was initiated 24 h after the most recent HIIT session (**Figure 1**). All OFT assessments were conducted at 10:00.

#### 2.4.2 Sucrose preference test

The sucrose preference test (SPT) was performed as previously described (Bekris et al., 2005; Dwivedi et al., 2015). Before group assignment for CUMS modeling, rats underwent sucrose habituation training to minimize novelty responses and side preference effects. Briefly, two bottles of 1% (w/v) sucrose solution were placed in each cage for 24 h to familiarize rats with sucrose. One sucrose bottle was then replaced with distilled water, and rats were given access to one bottle of sucrose solution and one bottle of distilled water for an additional 24 h. To reduce side bias, the positions of the two bottles were exchanged every 12 h during the habituation period. Sucrose habituation training was conducted during the final 2 days of the 1-week acclimation period. During the final 24 h of sucrose habituation, sucrose and water intake were recorded and sucrose preference was calculated to assess pre-CUMS comparability between the subsequently assigned Con and CUMS groups. Because these data were obtained during the 24-h habituation period, they were analyzed separately from the subsequent formal SPT assessments.

During the CUMS modeling period, the SPT was performed following each corresponding OFT assessment. During the HIIT intervention, two additional SPT assessments were conducted, followed by a final assessment after completion of the intervention. Each SPT was performed on the day following the corresponding OFT assessment (**Figure 1**). No additional food or water deprivation was imposed specifically before the SPT assessments. For each testing session, rats were individually housed and given access to two bottles for 3 h: one containing 100 mL of 1% (w/v) sucrose solution and the other containing 100 mL of distilled water. To minimize side bias, bottle positions were switched every 1.5 h. After the 3-h test, the amounts of sucrose solution and distilled water consumed were determined by weighing the bottles. Sucrose preference was calculated as sucrose intake (g) divided by total fluid intake (g) multiplied by 100%.

Behavioral outcome assessments, including OFT scoring and SPT fluid-intake measurements, were performed by investigators blinded to group allocation.

### 2.5 Sample collection

The prefrontal cortex, ileal tissue, and fecal samples were collected 12 h after completion of the final behavioral assessment to minimize potential confounding effects of test-induced stress. During this interval, all rats underwent the same 12-h food and water deprivation period as part of the standardized terminal sampling procedure. The deprivation procedure was applied identically across groups. Deprivation was initiated only after completion of all behavioral assessments. Rats from different groups were dissected in alternating order to ensure that sample collection occurred in a comparable time window across groups. The sampling procedure was as follows: 1) Rats were anesthetized with CO_2_ inhalation and euthanized by cervical dislocation, after which the whole brain was removed. 2) The prefrontal cortex was carefully dissected on ice by the same experimenter according to anatomical landmarks. Tissues were briefly rinsed with ice-cold 0.9% saline and gently blotted dry to minimize contamination during subsequent assays. 3) The prefrontal cortex samples were immediately transferred to cryogenic tubes, flash frozen in liquid nitrogen, and stored at -80 °C until analysis.

In parallel with brain dissection, a second experimenter collected ileal tissue. The ileal segment was carefully isolated, and the intestinal lumen was flushed with 0.9% saline to remove residual luminal contents before further processing. The ileal tissue was then gently blotted dry, aliquoted into 50-mg wet-weight portions, immediately flash-frozen in liquid nitrogen, and stored at -80 °C until analysis. Fresh fecal samples were collected from the distal rectum using sterile forceps and were likewise immediately flash-frozen in liquid nitrogen and stored at -80 °C. For 16S rRNA gene sequencing, three fecal samples were randomly selected from each group (n = 3 per group). Fecal samples for microbiota profiling were collected only at this terminal time point. No microbiota samples were collected before CUMS exposure or after CUMS and before the HIIT intervention.

### 2.6 ELISA measurements

#### 2.6.1 Tissue homogenization

For each tissue type, an equal wet tissue mass was used across samples and homogenized in ice-cold phosphate-buffered saline (PBS, pH 7.4) at a fixed tissue-to-buffer ratio of 1:9 (g/mL) using stainless steel beads. Homogenization was performed using a precooled low-temperature homogenizer at 4 °C for three cycles, with each cycle consisting of 30 s of homogenization followed by a 15-s pause. The homogenate was then centrifuged at 14,000 × *g* for 10 min at 4 °C. The supernatant from the 10% (w/v) homogenate was collected for ELISA.

#### 2.6.2 Assessment of LPS and IL-1β

LPS and IL-1β levels were measured using ELISA kits (Jiangsu Jingmei Biotechnology Co., Ltd., Yancheng, Jiangsu, China) according to the manufacturer’s instructions. For the LPS assay, the standard concentrations were 0, 50, 100, 200, 400, and 800 EU/L, with a manufacturer-specified detection range of 25–800 EU/L and a sensitivity of <1.0 EU/L. For the IL-1β assay, the standard concentrations were 0, 7.5, 15, 30, 60, and 120 pg/mL, with a detection range of 1–120 pg/mL and a sensitivity of <1.0 pg/mL. The intra- and inter-assay coefficients of variation were <15% for both assays. Briefly, reagents were brought to room temperature, and the wash buffer was prepared by diluting the concentrate 1:20 with distilled water. Standards (50 µL per well) were added to the designated standard wells, whereas sample wells received 10 µL of tissue homogenate supernatant followed by 40 µL of sample diluent. Horseradish peroxidase-conjugated detection antibody was then added at 100 µL per well. Standards and samples were assayed in duplicate. Plates were sealed and incubated at 37 °C for 60 min. Wells were then washed five times with wash buffer. Color development was initiated by adding substrate solutions A and B at 50 µL each per well and incubating the plate in the dark at 37 °C for 15 min. The reaction was terminated by adding 50 µL of stop solution per well. Absorbance was read at 450 nm using a microplate reader (Multiskan MS, type 352; Labsystems, Helsinki, Finland). Sample concentrations were calculated from the corresponding standard curves, and the values obtained from the standard curves were multiplied by five to correct for the fivefold sample dilution specified by the manufacturer. For LPS, dilution-corrected concentrations were expressed as EU/L of tissue-homogenate supernatant. For IL-1β, dilution-corrected concentrations were further normalized to tissue wet weight based on the 10% (w/v) tissue homogenate and expressed as pg/g tissue. All samples included in the analysis fell within the manufacturer-specified quantifiable range after accounting for the prescribed sample dilution. Because LPS was measured in different tissue matrices, prefrontal cortical and ileal LPS concentrations were analyzed and interpreted separately rather than compared directly between tissues.

### 2.7 Assessment of *TLR4* and *Rela* mRNA expression

Total RNA was extracted from the rat prefrontal cortex tissue using RnaEx™ Total RNA Isolation Solution (Shanghai Generay Biotech Co., Ltd., Shanghai, China). Briefly, the prefrontal cortex tissue was homogenized and lysed in the RnaEx reagent. Phase separation, RNA precipitation, and purification were then performed according to the manufacturer’s instructions, including a column purification step. The RNA was finally dissolved in DEPC-treated water, and the RNA concentration and purity were evaluated by measuring optical density values using a NanoDrop 5000 spectrophotometer (Thermo Fisher Scientific, USA).

Reverse transcription was performed using the HiScript^®^ II 1st Strand cDNA Synthesis Kit (Vazyme Biotech Co., Ltd., Nanjing, China). The first-strand cDNA synthesis reaction was prepared in an RNase-free microcentrifuge tube to a final volume of 15 μL, comprising 700 ng of total RNA, 2 μL of 5×HiScript^®^ II qRT SuperMix, and RNase-free ddH2O to volume. After gentle mixing, reverse transcription was carried out at 25 °C for 10 min, 50 °C for 30 min, and 85 °C for 5 min. The resulting cDNA was used immediately for qPCR or stored at -20 °C until use.

The mRNA expression levels of *TLR4* and *Rela*, the gene encoding the NF-κB p65/RelA subunit, were quantified by real-time quantitative PCR. Quantitative real-time PCR was performed on an ABI 7500 Real-Time PCR System (Thermo Fisher Scientific, Waltham, MA, USA) using the SYBR Green I method with Power qPCR PreMix (Shanghai Generay Biotech Co., Ltd., Shanghai, China). Each sample was assayed in three technical replicates. Reactions were prepared in 96-well plates with a total volume of 16 μL per well containing 7 μL SYBR Green Mix, 0.5 μL forward primer, 0.5 μL reverse primer, and 8 μL cDNA. The thermal cycling conditions were 95 °C for 10 min, followed by 40 cycles of 95 °C for 10 s and 60 °C for 34 s. A melting curve analysis was performed to verify amplification specificity using 95 °C for 15 s, 60 °C for 60 s, 95 °C for 30 s, and 60 °C for 15 s. Raw qPCR data were exported from the ABI software and analyzed using DataAssist software. The Ct values of the target genes (*TLR4* and *Rela*) were normalized to the reference gene (*GAPDH*), and the relative mRNA expression levels were calculated using the 2^-ΔΔCt^ method.

The primer sequences (5′ to 3′) used were as follows: *GAPDH* forward, AGGTCGGTGTGAACGGGTGAG; *GAPDH* reverse, GGGGTCGTTGATGGCAACA; *TLR4* forward, GCCAGAATGAGGACTGGGTGA; *TLR4* reverse, GTTGGCAGCAATGGCTACACC; *Rela* forward, TTCAATGGACCAACTGAACCC; *Rela* reverse, GGATGTTGAAAAGGCATAGGG.

### 2.8 Gut microbiota profiling

To characterize gut microbiota profiles across the experimental groups, microbial DNA was extracted from rat fecal samples using the MN NucleoSpin 96 Soil kit. The V4 hypervariable region of the 16S rRNA gene was selected as the amplification target, and PCR amplification was performed using primers 515F (5′-GTGYCAGCMGCCGCGGTAA-3′) and 806R2 (5′-GGACTACNVGGGTWTCTAAT-3′). Amplicons were purified, sequencing libraries were constructed, and paired-end sequencing was performed on the Illumina NovaSeq platform. A total of 12 samples were sequenced, yielding 840,000 raw read pairs. After read merging with FLASH (version 1.2.11), quality filtering with Trimmomatic (version 0.33), and chimera removal with UCHIME (version 8.1), 825,272 high-quality sequences were retained. Each sample contributed at least 68,532 sequences, with a mean of 68,773 sequences per sample, and the mean sequence length was 252 bp. High-quality sequences were then clustered into operational taxonomic units (OTUs) at a 97% sequence similarity threshold, resulting in 668 OTUs. The number of OTUs per sample ranged from 595 to 628. Rarefaction curves approached a plateau, indicating adequate sequencing depth for the included samples. Taxonomic annotation of representative OTU sequences was originally performed using the SILVA 138 database. Taxonomic nomenclature was reviewed and harmonized according to SILVA release 138.2 when an unambiguous nomenclatural correspondence was available. This procedure involved nomenclature harmonization rather than sequence-level re-annotation. At the phylum level, taxa corresponding to the same updated taxonomic category were combined before recalculation of relative abundance and subsequent statistical analyses. Taxa that could not be reliably assigned to lower taxonomic ranks were retained at the highest confidently assigned level. Database-specific phylogenetic group labels were retained when no unambiguous updated taxonomic assignment was available.

Gut microbiota analyses included OTU richness, alpha diversity, beta diversity, and relative abundance at the phylum and genus levels. Alpha diversity was assessed using the ACE, Chao1, Simpson, and Shannon indices. The ACE and Chao1 indices were used to estimate community richness, with higher values indicating greater richness. The Simpson index, expressed as 1-D, and the Shannon index were used to evaluate both richness and evenness, with higher values indicating greater community diversity. Beta diversity was evaluated using weighted UniFrac distances, and non-metric multidimensional scaling (NMDS) was used to visualize differences in gut microbial community composition across samples. Group separation was assessed using analysis of similarities (ANOSIM) based on the weighted UniFrac distance matrix. Smaller weighted UniFrac distances indicate greater similarity in community composition.

Taxonomic composition was analyzed at the phylum and genus levels. At each taxonomic level, the 10 most abundant taxa based on mean relative abundance across samples were selected for stacked bar plots, and taxa outside the top 10 were merged as “Others.”

### 2.9 Statistical analysis

Unless otherwise specified, quantitative data are presented as mean ± SEM. During the CUMS modeling phase, longitudinal body-weight, SPT, and OFT outcomes were analyzed using mixed-design repeated-measures ANOVAs, with CUMS exposure as the between-subject factor and test as the within-subject factor. Pre-CUMS sucrose-preference data obtained during the final day of sucrose habituation were analyzed separately using an independent-samples *t* test. During the HIIT intervention phase, body-weight, SPT, and OFT outcomes were analyzed using three-way mixed-design repeated-measures ANOVAs, with CUMS exposure and exercise as between-subject factors and time for body weight or test for behavioral outcomes as the within-subject factor. Longitudinal repeated-measures analyses were performed using complete cases across the included time points. Vmax was analyzed using a two-way mixed-design repeated-measures ANOVA, with group as the between-subject factor and time as the within-subject factor. Biochemical outcomes and alpha-diversity indices were analyzed using two-way ANOVAs, with CUMS exposure and exercise as between-subject factors. When significant interactions were detected, simple-effects analyses were performed. Effect sizes are reported as partial eta squared (ηp²) for ANOVA effects and Cohen’s *d* for independent-samples *t* tests. Mean differences with 95% confidence intervals are reported for relevant simple-effects comparisons. For repeated-measures analyses involving more than two time points, Greenhouse-Geisser correction was applied when the assumption of sphericity was violated. Fisher’s least significant difference (LSD) *post hoc* comparisons were performed when applicable. Statistical significance was set at *P* < 0.05.

For gut microbiota analyses, group separation in beta diversity was assessed using analysis of similarities (ANOSIM) based on weighted UniFrac distances. Linear discriminant analysis effect size (LEfSe) was used as an exploratory screening approach to identify candidate differential taxa among the four groups, with thresholds of *P* < 0.05 and an LDA score > 2.0. For each LEfSe-identified candidate taxon, Dunn’s *post hoc* tests were performed for all six pairwise comparisons among the four groups, with Benjamini-Hochberg adjustment applied to the six comparisons within each taxon. Three biologically relevant comparisons were emphasized: Con_Sed versus CUMS_Sed, Con_Sed versus Con_Ex, and CUMS_Sed versus CUMS_Ex. Candidate taxa with an adjusted *P* < 0.05 in at least one of these three comparisons were displayed in the pairwise-comparison heatmaps. For visualization, the sign of the Dunn z statistic was oriented according to the mean-rank difference between the two groups, with positive values indicating a higher mean rank in the first group and negative values indicating a higher mean rank in the second group. Significance symbols represent BH-adjusted *P* values. The same taxa were also displayed across individual biological samples using row-wise Z-score-transformed relative abundance values. The Z-score transformation was used only for visualization and did not affect the statistical analyses. Given the limited biological replication, microbiota analyses, particularly taxon-level comparisons, were treated as exploratory. The data were analyzed using SPSS Statistics version 27.0 (IBM Corp., Armonk, NY, USA) and R version 4.3.3 when appropriate. Figures were generated using GraphPad Prism 9 (La Jolla, CA, USA), and schematic illustrations were created with BioRender.com.

## 3 Results

### 3.1 CUMS induced depressive-like behaviors

To characterize physiological and behavioral changes following CUMS exposure before the HIIT intervention, body weight was monitored as a secondary physiological outcome, and depressive-like behaviors were assessed using the SPT and OFT. Body weight was analyzed across weeks 0-4, with week 0 representing the acclimation period.

For body weight, there were significant main effects of CUMS (Con and CUMS; *F*(1, 38) = 175.763, *P* < 0.001, ηp^2^ = 0.822) and time (weeks 0-4; *F*(1.82, 69.16) = 130.972, *P* < 0.001, ηp^2^ = 0.775), as well as a significant CUMS × time interaction (*F*(1.82, 69.16) = 165.746, *P* < 0.001, ηp^2^ = 0.813). Given the significant CUMS × time interaction, simple-effects analyses showed that body weight did not differ significantly between the Con and CUMS groups at week 0 (*P* = 0.168), whereas body weight was significantly lower in the CUMS group than in the Con group during weeks 1-4 (all *P* < 0.001; **Figure 2A**).

**Figure 2:**
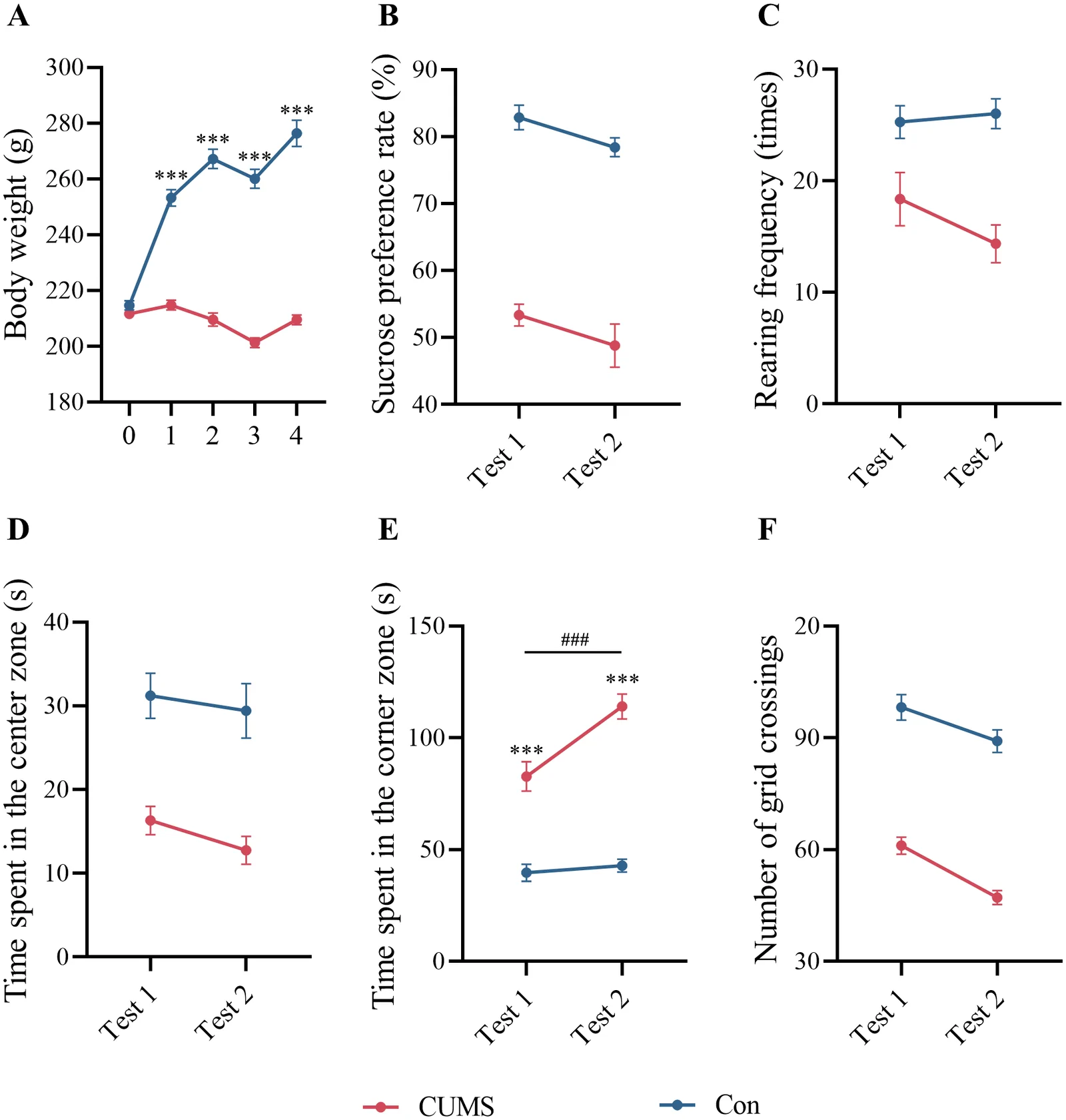
Body weight, sucrose preference test, and open field test outcomes before the HIIT intervention. **(A)** Changes in body weight during weeks 0–4 in the Con and CUMS groups. Week 0 corresponds to the acclimation period. **(B)** Sucrose preference at the two formal SPT assessments. **(C-F)** OFT outcomes at the two assessments, including rearing frequency **(C)**, time spent in the center zone **(D)**, time spent in the corner zone **(E)**, and number of grid crossings **(F)**. Data are presented as mean ± SEM (n = 20 per group) and were analyzed using two-way mixed-design repeated-measures ANOVA, followed by simple-effects analyses when significant interactions were detected. ^***^P < 0.001 for CUMS versus Con at the corresponding assessment; ^###^*P* < 0.001 for Test 2 versus Test 1 within the CUMS group. SPT, sucrose preference test; OFT, open field test; HIIT, high-intensity interval training; CUMS, chronic unpredictable mild stress; Con, control.

For the SPT, pre-CUMS sucrose preference recorded during the final day of sucrose habituation did not differ significantly between rats subsequently assigned to the Con and CUMS groups (*t*(38) = -0.556, *P* = 0.581, Cohen’s *d* = -0.176; **Supplementary Figure 1**). For the two subsequent formal SPT assessments, there were significant main effects of CUMS exposure (*F*(1, 38) = 164.549, *P* < 0.001, ηp^2^ = 0.812) and test (*F*(1, 38) = 5.166, *P* = 0.029, ηp^2^ = 0.120), whereas the CUMS × test interaction was not significant (*F*(1, 38) = 0.001, *P* = 0.980, ηp^2^ < 0.001). Across the two assessments, sucrose preference was lower in CUMS rats than in control rats, and sucrose preference differed significantly between the two assessments (**Figure 2B**).

For the OFT, rearing frequency showed a significant main effect of CUMS exposure (*F*(1, 38) = 18.974, *P* < 0.001, ηp^2^ = 0.333), whereas neither the main effect of test (*F*(1, 38) = 1.548, *P* = 0.221, ηp^2^ = 0.039) nor the CUMS × test interaction (*F*(1, 38) = 3.315, *P* = 0.077, ηp^2^ = 0.080) was significant. Across both assessments, rearing frequency was lower in the CUMS group than in the Con group (**Figure 2C**).

Similarly, time spent in the center zone showed a significant main effect of CUMS exposure (*F*(1, 38) = 35.473, *P* < 0.001, ηp^2^ = 0.483), whereas neither the main effect of test (*F*(1, 38) = 1.532, *P* = 0.223, ηp^2^ = 0.039) nor the CUMS × test interaction (*F*(1, 38) = 0.164, *P* = 0.688, ηp^2^ = 0.004) was significant. Across both assessments, CUMS rats spent significantly less time in the center zone than control rats (**Figure 2D**).

For time spent in the corner zone, there were significant main effects of CUMS exposure (*F*(1, 38) = 141.374, *P* < 0.001, ηp^2^ = 0.788) and test (*F*(1, 38) = 11.774, *P* = 0.001, ηp^2^ = 0.237), as well as a significant CUMS × test interaction (*F*(1, 38) = 7.859, *P* = 0.008, ηp^2^ = 0.171). Simple-effects analyses showed that CUMS rats spent significantly more time in the corner zone than control rats at both test 1 and test 2 (both *P* < 0.001). Within the CUMS group, corner-zone duration was significantly greater at test 2 than at test 1 (*P* < 0.001), whereas no significant difference between the two assessments was observed in the control group (*P* = 0.660; **Figure 2E**).

For the number of grid crossings, there were significant main effects of CUMS exposure (*F*(1, 38) = 178.104, *P* < 0.001, ηp^2^ = 0.824) and test (*F*(1, 38) = 22.189, *P* < 0.001, ηp^2^ = 0.369), whereas the CUMS × test interaction was not significant (*F*(1, 38) = 0.966, *P* = 0.332, ηp^2^ = 0.025). Across both tests, grid crossings were lower in the CUMS group than in the Con group, and the number of grid crossings differed significantly between the two assessments (**Figure 2F**). Detailed simple-effects estimates and 95% confidence intervals for body weight and corner-zone duration, which showed significant interactions, are provided in **Supplementary Table 1**.

### 3.2 HIIT increased maximal running speed and partially attenuated CUMS-induced depressive-like behaviors

Vmax increased significantly over the first 2 weeks of HIIT. Mixed-design repeated-measures ANOVA revealed a significant main effect of time (pre-HIIT and mid-HIIT; *F*(1, 18) = 72.000, *P* < 0.001, ηp^2^ = 0.800), whereas neither the main effect of group (Con_Ex and CUMS_Ex; *F*(1, 18) = 0.900, *P* = 0.355, ηp^2^ = 0.048) nor the group × time interaction (*F*(1, 18) = 0.000, *P* = 1.000, ηp^2^ = 0.000) was significant. Pairwise comparisons indicated that Vmax did not differ significantly between the Con_Ex and CUMS_Ex groups either before HIIT (*P* = 0.433) or after 2 weeks of training (*P* = 0.310; **Supplementary Figure 2**).

One rat in the CUMS_Ex group died during week 3 of the HIIT intervention. The cause of death could not be definitively established. Outcomes assessed across the full HIIT intervention and post-intervention biochemical outcomes therefore included nine rats in the CUMS_Ex group. For body weight, there was a significant main effect of CUMS exposure (*F*(1, 35) = 29.899, *P* < 0.001, ηp^2^ = 0.461), whereas the main effect of exercise was not significant. The main effect of time (weeks 5-9; *F*(2.435, 85.241) = 195.694, *P* < 0.001, ηp^2^ = 0.848), CUMS × time interaction (*F*(2.435, 85.241) = 30.435, *P* < 0.001, ηp^2^ = 0.465), and exercise × time interaction (*F*(2.435, 85.241) = 44.918, *P* < 0.001, ηp^2^ = 0.562) were significant, whereas the CUMS × exercise and CUMS × exercise × time interactions were not. Simple-effects analyses of the CUMS × time interaction showed that, averaged across exercise conditions, body weight was significantly lower in CUMS rats than in control rats at all five assessments (weeks 5-8, all *P* < 0.001; week 9, *P* = 0.002). Simple-effects analyses of the exercise × time interaction showed that, averaged across CUMS conditions, body weight did not differ significantly between exercised and sedentary rats at weeks 5-7 (*P* = 0.339, 0.738, and 0.596, respectively), whereas body weight was significantly lower in exercised rats at weeks 8 and 9 (*P* = 0.008 and 0.009, respectively; **Figure 3A**).

**Figure 3:**
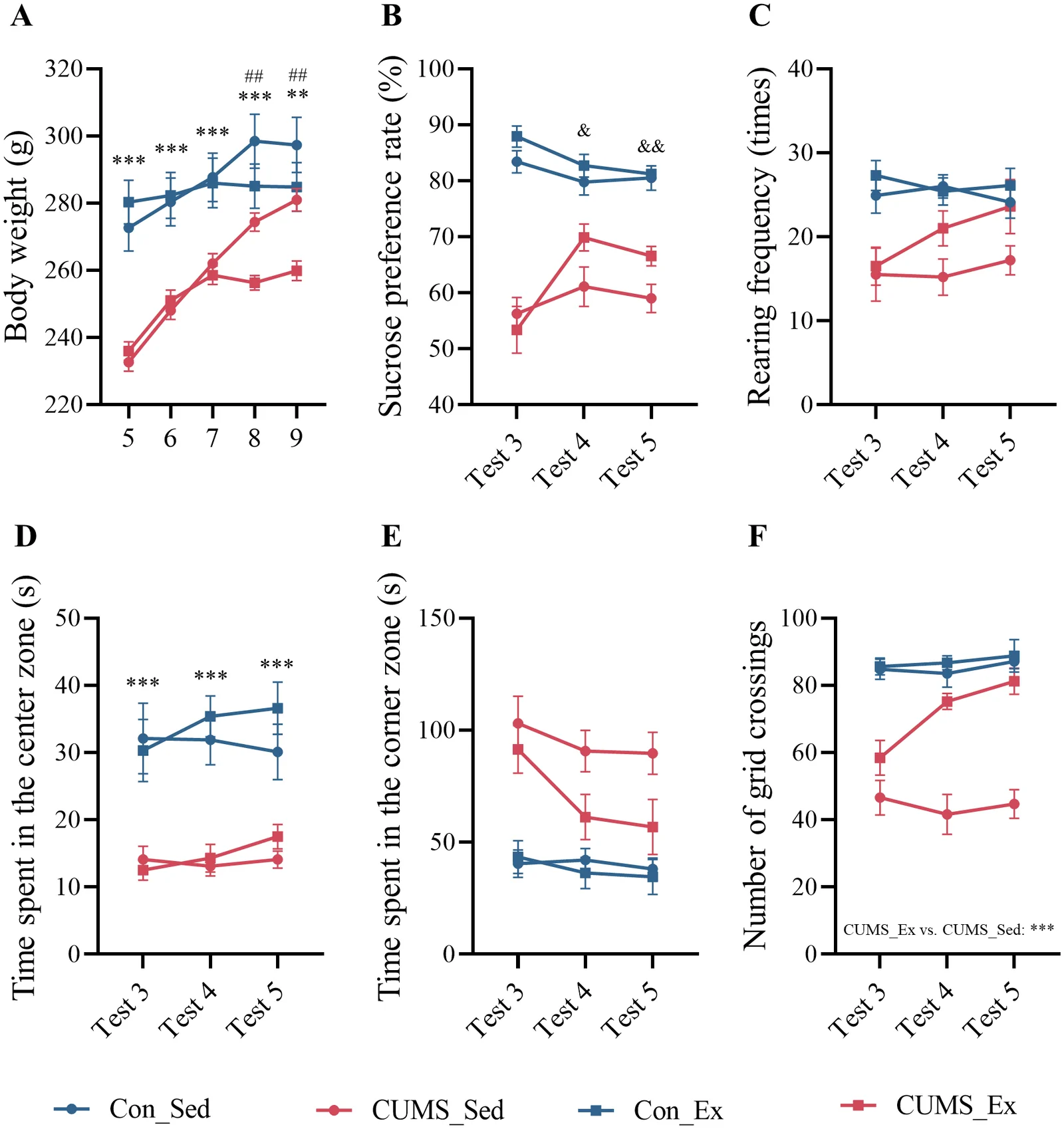
Body weight, sucrose preference test, and open field test outcomes during and after the HIIT intervention. **(A)** Changes in body weight during weeks 5–9 in the Con_Sed, Con_Ex, CUMS_Sed, and CUMS_Ex groups. **(B)** Sucrose preference across tests 3-5. **(C-F)** OFT outcomes across tests 3-5, including rearing frequency **(C)**, time spent in the center zone **(D)**, time spent in the corner zone **(E)**, and number of grid crossings **(F)**. The center zone comprised the central nine squares and the corner zone comprised the four corner squares. The remaining 12 squares were not included in either zone-duration measure. Each OFT was conducted 24 h after the most recent HIIT session and 24 h before the corresponding SPT. Data are presented as mean ± SEM and were analyzed using three-way mixed-design repeated-measures ANOVA, with CUMS exposure and exercise as between-subject factors and time (for body weight) or test (for behavioral outcomes) as the within-subject factor. For the repeated-measures analyses, complete cases were used, with n = 9 in the CUMS_Ex group and n = 10 in each of the other three groups across all assessments. For **(A)**, ^**^P < 0.01, and ^***^P < 0.001 indicate the simple effect of CUMS exposure at the corresponding assessment, averaged across exercise conditions, and ^##^*P* < 0.01 indicates the simple effect of exercise, averaged across CUMS conditions. For **(B)**, ^&^*P* < 0.05 and ^&&^*P* < 0.01 indicate CUMS_Ex versus CUMS_Sed at the corresponding assessment. For **(D)**, ^***^P < 0.001 indicates the simple effect of CUMS exposure at the corresponding assessment, averaged across exercise conditions. For **(F)**, ^***^P < 0.001 indicates that simple-effects analysis of the CUMS × exercise interaction showed significantly higher grid crossings in exercised than sedentary CUMS rats when averaged across assessments, whereas no significant exercise effect was observed in control rats (*P* = 0.529). SPT, sucrose preference test; OFT, open field test; HIIT, high-intensity interval training; CUMS_Sed, chronic unpredictable mild stress sedentary; CUMS_Ex, chronic unpredictable mild stress exercise; Con_Sed, control sedentary; Con_Ex, control exercise.

For the SPT, there were significant main effects of CUMS exposure (*F*(1, 35) = 164.088, *P* < 0.001, ηp^2^ = 0.824) and exercise (*F*(1, 35) = 4.472, *P* = 0.042, ηp^2^ = 0.113), whereas the main effect of test was not significant. A significant CUMS × test interaction was observed (*F*(2, 70) = 12.771, *P* < 0.001, ηp^2^ = 0.267), and the CUMS × exercise × test interaction was also statistically significant (*F*(2, 70) = 3.128, *P* = 0.04997, ηp² = 0.082). Simple-effects analyses of the three-way interaction showed that sucrose preference was significantly higher in the CUMS_Ex group than in the CUMS_Sed group at tests 4 (*P* = 0.027) and 5 (*P* = 0.009), but not at test 3 (*P* = 0.459). No significant exercise-related differences were observed in control rats at any assessment (all *P* > 0.05; **Figure 3B**).

For rearing frequency in the OFT, there was a significant main effect of CUMS exposure (*F*(1, 35) = 22.444, *P* < 0.001, ηp^2^ = 0.391), whereas the main effect of exercise was not significant (*F*(1, 35) = 3.380, *P* = 0.074, ηp^2^ = 0.088), nor was the CUMS × exercise interaction (*F*(1, 35) = 1.060, *P* = 0.310, ηp^2^ = 0.029). The main effect of test (*F*(1.548, 54.192) = 0.777, *P* = 0.435, ηp^2^ = 0.022) and the CUMS × test, exercise × test, and CUMS × exercise × test interactions were also not significant (all *P* > 0.05). Across exercise conditions and assessments, rearing frequency was lower in CUMS rats than in control rats (**Figure 3C**).

For time spent in the center zone, there was a significant main effect of CUMS exposure (*F*(1, 35) = 32.910, *P* < 0.001, ηp^2^ = 0.485), whereas the main effect of exercise was not significant (*F*(1, 35) = 0.355, *P* = 0.555, ηp^2^ = 0.010). There was also a significant main effect of test (*F*(1.107, 38.736) = 13.361, *P* < 0.001, ηp^2^ = 0.276), together with significant CUMS × test (*F*(1.107, 38.736) = 4.573, *P* = 0.035, ηp^2^ = 0.116) and exercise × test interactions (*F*(1.107, 38.736) = 27.831, *P* < 0.001, ηp^2^ = 0.443). The CUMS × exercise interaction and CUMS × exercise × test interaction were not significant. Simple-effects analyses of the CUMS × test interaction showed that, averaged across exercise conditions, CUMS rats spent significantly less time in the center zone than control rats at tests 3-5 (all *P* < 0.001). For the exercise × test interaction, simple-effects analyses showed no significant differences between exercised and sedentary rats at tests 3–5 when averaged across CUMS conditions (all *P* > 0.05; **Figure 3D**).

For time spent in the corner zone, there were significant main effects of CUMS exposure (*F*(1, 35) = 39.991, *P* < 0.001, ηp^2^ = 0.533) and test (*F*(1.202, 42.054) = 6.313, *P* = 0.012, ηp^2^ = 0.153), whereas the main effect of exercise and all interaction effects were not significant. CUMS rats spent more time in the corner zone than control rats, and corner-zone duration differed significantly across the three assessments (**Figure 3E**).

For the number of grid crossings, there were significant main effects of CUMS exposure (*F*(1, 35) = 171.970, *P* < 0.001, ηp^2^ = 0.831) and exercise (*F*(1, 35) = 46.457, *P* < 0.001, ηp^2^ = 0.570), as well as a significant CUMS × exercise interaction (*F*(1, 35) = 35.163, *P* < 0.001, ηp^2^ = 0.501). The main effect of test and all interactions involving test were not significant (all *P* > 0.05). Simple-effects analyses of the CUMS × exercise interaction showed that, averaged across assessments, grid crossings were significantly higher in exercised than sedentary CUMS rats (*P* < 0.001), whereas no significant exercise effect was observed in control rats (*P* = 0.529; **Figure 3F**). Detailed simple-effects estimates and 95% confidence intervals for outcomes showing significant interactions are provided in **Supplementary Table 2**.

### 3.3 HIIT was associated with differences in gut microbial diversity and composition

#### 3.3.1 OTU richness

To characterize group differences in OTU-level microbial profiles, OTU richness was first examined. Following 16S rRNA gene sequencing and quality control, sequences were clustered into OTUs at a 97% similarity threshold, yielding 668 OTUs in total. The number of OTUs per sample ranged from 595 to 628. Rarefaction curves approached a plateau, indicating that the sequencing depth was sufficient to capture most detectable taxa across all samples (**Supplementary Figure 3**). A Venn diagram revealed that 623 OTUs were shared among the four groups. Additionally, Con_Ex and CUMS_Ex contained 1 and 2 unique OTUs, respectively (**Figure 4A**).

**Figure 4:**
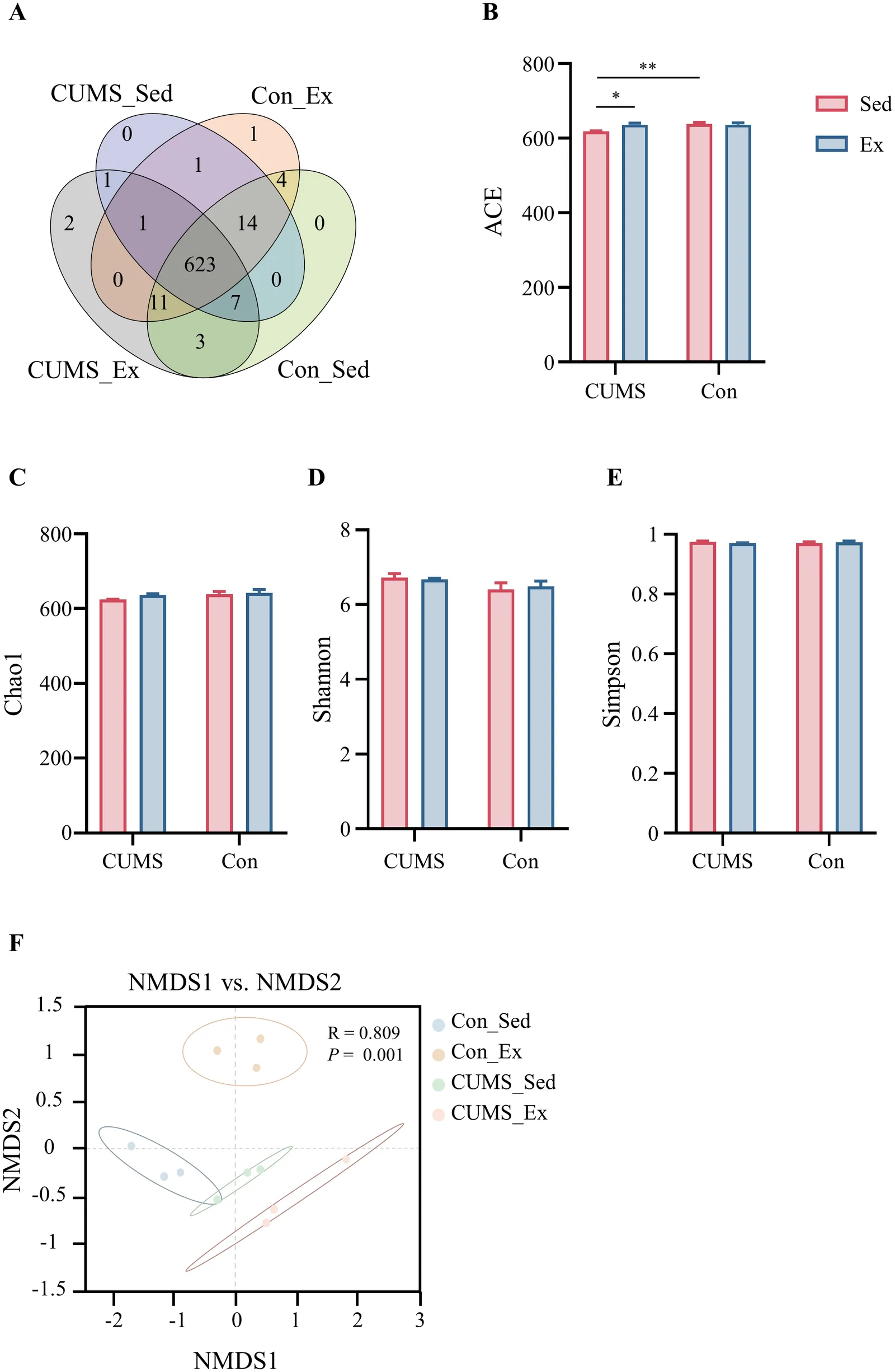
Gut microbiota richness, alpha diversity, and beta diversity across groups. **(A)** Venn diagram of shared and unique OTUs among the Con_Sed, Con_Ex, CUMS_Sed, and CUMS_Ex groups, with OTUs defined at 97% sequence similarity. **(B-E)** Alpha diversity indices, including ACE, Chao1, Shannon, and Simpson (1-D), in each group. **(F)** NMDS ordination of gut microbial community structure based on weighted UniFrac distances. Each point represents an individual sample. Group separation was assessed using ANOSIM based on the weighted UniFrac distance matrix, yielding R = 0.809 and *P* = 0.001. Alpha diversity indices are presented as mean ± SEM (n = 3 per group) and were analyzed using a two-way ANOVA, followed by simple-effects analyses when significant interactions were detected. ^*^P < 0.05, ^**^P < 0.01 for indicated comparisons. OTUs, operational taxonomic units; NMDS, non-metric multidimensional scaling; ANOSIM, analysis of similarities.

#### 3.3.2 Diversity of the gut microbiota

To further characterize group differences in within-sample microbial diversity and between-sample community structure, alpha and beta diversity analyses were performed. For alpha diversity, the ACE index exhibited a significant main effect of CUMS exposure (CUMS and Con; *F*(1, 8) = 6.298, *P* = 0.036, ηp^2^ = 0.44) and a significant CUMS × exercise interaction (*F*(1, 8) = 5.848, *P* = 0.042, ηp^2^ = 0.422), whereas the main effect of exercise was not significant (*F*(1, 8) = 4.57, *P* = 0.065, ηp^2^ = 0.364). Simple-effects analyses of the CUMS × exercise interaction showed that ACE values were lower in CUMS rats than in control rats under sedentary conditions (*P* = 0.008), but not under exercise conditions (*P* = 0.950). ACE values were higher in the CUMS_Ex group than in the CUMS_Sed group (*P* = 0.012), whereas no significant difference was observed between the Con_Ex and Con_Sed groups (*P* = 0.848; **Figure 4B**). No significant main effects or interactions were observed for the Chao1, Shannon, or Simpson indices (all *P* > 0.05; **Figures 4C–E**).

For beta diversity, the NMDS ordination plot showed apparent separation among the experimental groups (**Figure 4F**). The stress value was 0.0643, indicating a good representation of the original distance relationships. ANOSIM further indicated that between-group differences in gut microbial community structure were significantly greater than within-group differences (R = 0.809, *P* = 0.001).

#### 3.3.3 Taxonomic composition of the gut microbiota

To further characterize group differences in gut microbiota composition, community composition was analyzed at the phylum and genus levels. Based on taxonomic annotation, the 10 most abundant taxa at each taxonomic level were selected to generate stacked bar plots of relative abundance. At the phylum level, the gut microbiota across the four groups was dominated primarily by Bacillota, Bacteroidota, Pseudomonadota, and Actinomycetota (**Figure 5A**). At the genus level, the most abundant genus-level taxa included unclassified genus (UG) within Muribaculaceae, *Lactobacillus*, *Alloprevotella*, and *Dubosiella* (**Figure 5B**).

**Figure 5:**
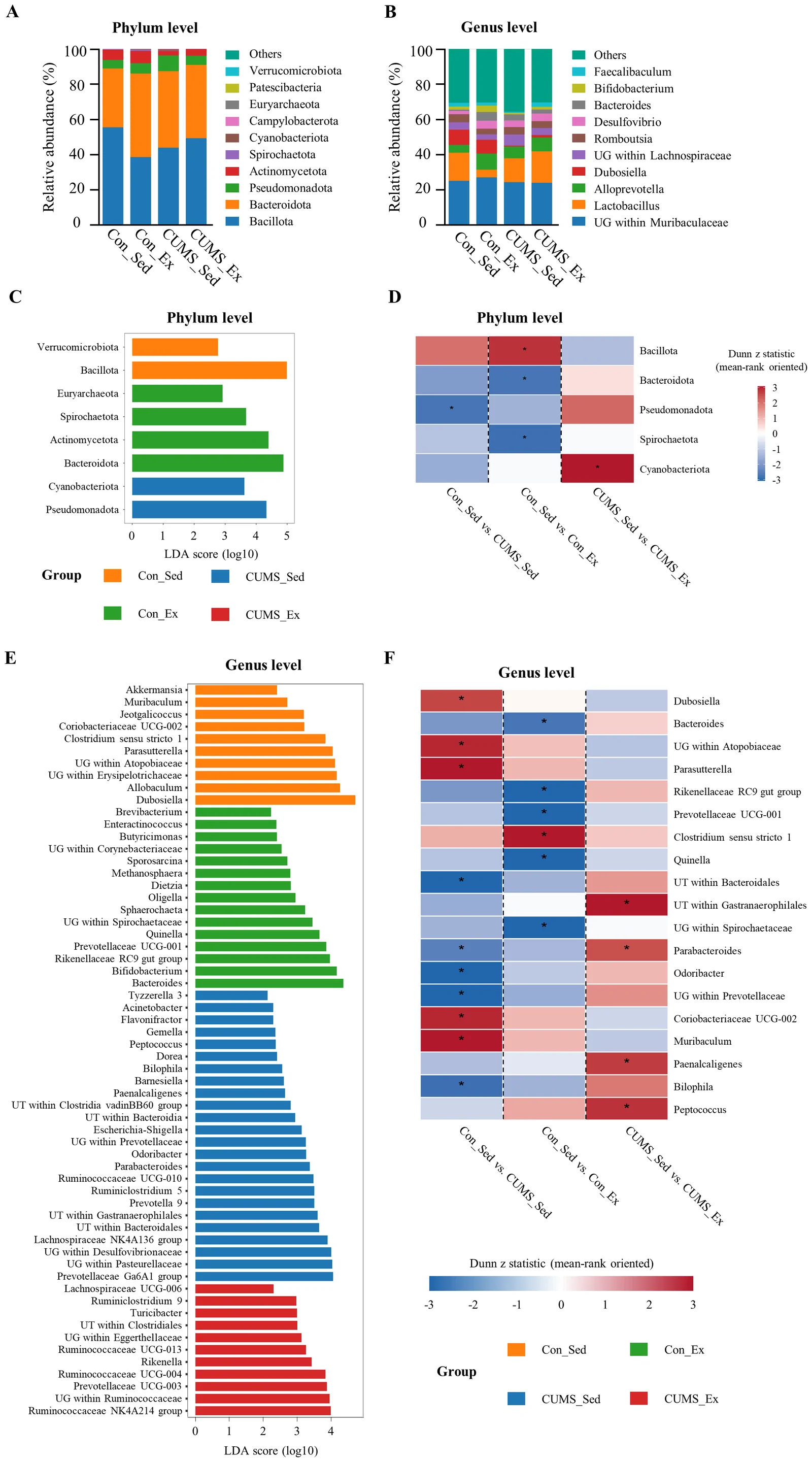
Gut microbiota composition and LEfSe-identified candidate discriminative taxa at the phylum and genus levels. **(A, B)** Stacked bar plots of the relative abundance of the top 10 phylum- and genus-level taxa across the Con_Sed, Con_Ex, CUMS_Sed, and CUMS_Ex groups. Taxa outside the top 10 were merged as “Others.” **(C)** LEfSe-identified candidate discriminative phylum-level taxa among the four groups. **(D)** Dunn’s *post hoc* pairwise-comparison heatmap for LEfSe-identified candidate phylum-level taxa with a BH-adjusted *P* < 0.05 in at least one of the three emphasized comparisons. **(E)** LEfSe-identified candidate discriminative genus-level taxa among the four groups. In **(C, E)**, bar length represents the LDA score, and bar color indicates the enriched group. **(F)** Dunn’s *post hoc* pairwise-comparison heatmap for LEfSe-identified candidate genus-level taxa with a BH-adjusted *P* < 0.05 in at least one of the three emphasized comparisons. The three emphasized comparisons were Con_Sed versus CUMS_Sed, Con_Sed versus Con_Ex, and CUMS_Sed versus CUMS_Ex. In **(D, F)**, the sign of the Dunn z statistic was oriented according to the mean-rank difference between the two groups for visualization. Positive values indicate a higher mean rank in the first-listed group, whereas negative values indicate a higher mean rank in the second-listed group. ^*^BH-adjusted *P* < 0.05, ^**^BH-adjusted *P* < 0.01. LEfSe, linear discriminant analysis effect size; LDA, linear discriminant analysis; UG, unclassified genus; UT, unclassified taxon.

Exploratory LEfSe analysis identified eight candidate discriminative phylum-level taxa among the four groups. Verrucomicrobiota and Bacillota were enriched in the Con_Sed group, Pseudomonadota and Cyanobacteriota were enriched in the CUMS_Sed group, whereas Bacteroidota, Actinomycetota, Spirochaetota, and Euryarchaeota were enriched in the Con_Ex group. No phylum-level taxon met the LEfSe criteria for enrichment in the CUMS_Ex group (**Figure 5C**).

For these LEfSe-identified candidate phyla, Dunn’s *post hoc* pairwise comparisons were performed with Benjamini-Hochberg adjustment applied to the six pairwise comparisons within each phylum. Among the three biologically relevant comparisons, the Con_Sed group showed a lower relative abundance of Pseudomonadota than the CUMS_Sed group (adjusted *P* = 0.039). Under non-stressed conditions, the Con_Sed group showed a higher relative abundance of Bacillota (adjusted *P* = 0.028) but lower relative abundances of Bacteroidota (adjusted *P* = 0.039) and Spirochaetota (adjusted *P* = 0.028) than the Con_Ex group. Under CUMS conditions, the CUMS_Sed group showed a higher relative abundance of Cyanobacteriota than the CUMS_Ex group (adjusted *P* = 0.013; **Figure 5D**). The individual-sample heatmap further illustrated the relative-abundance patterns of these phyla across biological replicates (**Supplementary Figure 4A**).

At the genus level, exploratory LEfSe analysis identified 60 candidate discriminative genus-level taxa among the four groups. Representative enriched taxa included *Dubosiella* and *Allobaculum* in the Con_Sed group, *Bacteroides* and *Bifidobacterium* in the Con_Ex group, Prevotellaceae Ga6A1 group and UG within Pasteurellaceae in the CUMS_Sed group, and Ruminococcaceae NK4A214 group and UG within Ruminococcaceae in the CUMS_Ex group (**Figure 5E**).

For these LEfSe-identified candidate taxa, Dunn’s *post hoc* pairwise comparisons were performed, with Benjamini-Hochberg adjustment applied to the six pairwise comparisons within each taxon. Compared with the CUMS_Sed group, the Con_Sed group showed higher relative abundances of *Muribaculum* (adjusted *P* = 0.013), *Parasutterella* (adjusted *P* = 0.013), Coriobacteriaceae UCG-002 (adjusted *P* = 0.019), UG within Atopobiaceae (adjusted *P* = 0.019), and *Dubosiella* (adjusted *P* = 0.038), but lower relative abundances of *Odoribacter* (adjusted *P* = 0.013), UG within Prevotellaceae (adjusted *P* = 0.013), unclassified taxon (UT) within Bacteroidales (adjusted *P* = 0.013), *Bilophila* (adjusted *P* = 0.028), and *Parabacteroides* (adjusted *P* = 0.038).

Under non-stressed conditions, compared with the Con_Ex group, the Con_Sed group showed lower relative abundances of Rikenellaceae RC9 gut group, Prevotellaceae UCG-001, *Quinella*, and UG within Spirochaetaceae (all adjusted *P* = 0.013), and *Bacteroides* (adjusted *P* = 0.039), whereas *Clostridium sensu stricto 1* was higher (adjusted *P* = 0.013).

Under CUMS conditions, compared with the CUMS_Ex group, the CUMS_Sed group showed higher relative abundances of *Peptococcus* (adjusted *P* = 0.028), UT within Gastranaerophilales (adjusted *P* = 0.013), *Parabacteroides* (adjusted *P* = 0.038), and *Paenalcaligenes* (adjusted *P* = 0.039; **Figure 5F**). The individual-sample heatmap further illustrated the relative-abundance patterns of these genus-level taxa across biological replicates (**Supplementary Figure 4B**).

### 3.4 HIIT was associated with lower prefrontal cortical LPS and inflammation-related markers in CUMS rats

To investigate whether exercise was associated with changes in LPS-related inflammatory markers in CUMS rats, LPS and IL-1β levels and *TLR4* and *Rela* mRNA expression were assessed in the prefrontal cortex, while LPS levels were measured in ileal tissue.

For prefrontal cortical LPS levels, there were significant main effects of CUMS exposure (*F*(1, 35) = 27.278, *P* < 0.001, ηp^2^ = 0.438) and exercise (*F*(1, 35) = 6.881, *P* = 0.013, ηp^2^ = 0.164), as well as a significant CUMS × exercise interaction (*F*(1, 35) = 5.795, *P* = 0.021, ηp^2^ = 0.142). Simple-effects analyses of the CUMS × exercise interaction showed that, under sedentary conditions, prefrontal cortical LPS levels were significantly higher in CUMS rats than in control rats (*P* < 0.001), whereas this difference was not statistically significant under exercise conditions (*P* = 0.057). Within CUMS rats, exercise was associated with significantly lower prefrontal cortical LPS levels compared with sedentary conditions (*P* = 0.001), whereas no significant exercise-related difference was observed in control rats (*P* = 0.878; **Figure 6A**).

**Figure 6:**
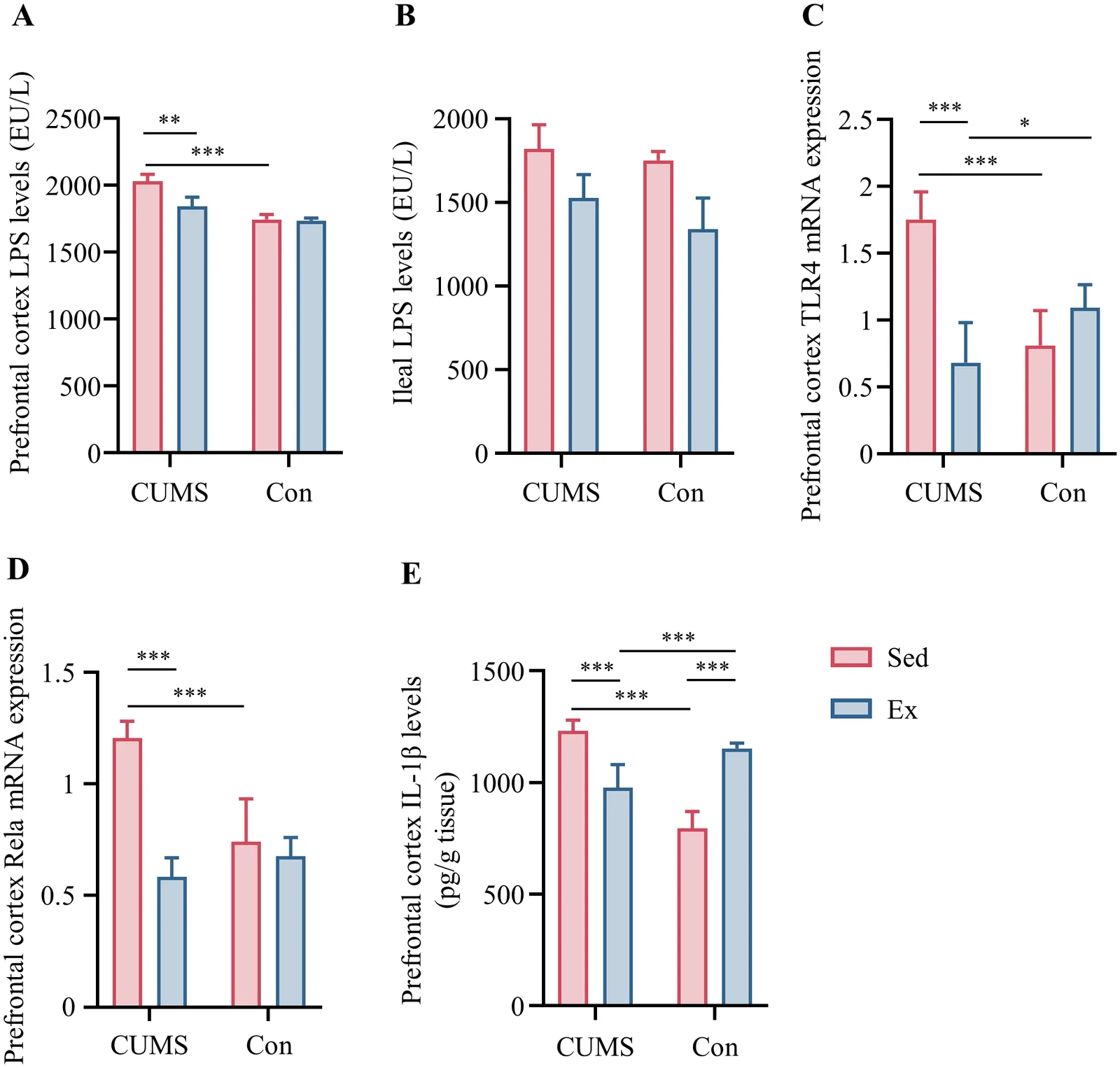
Effects of HIIT on LPS-associated inflammatory measures and *TLR4/Rela* mRNA expression. **(A)** Prefrontal cortical LPS levels. **(B)** Ileal LPS levels. **(C)** Prefrontal cortical *TLR4* mRNA expression. **(D)** Prefrontal cortical *Rela* mRNA expression. **(E)** Prefrontal cortical IL-1β levels. Sample sizes were n = 10 per group, except for the CUMS_Ex group (n = 9). For **(B)**, the CUMS × exercise interaction was not significant, whereas a significant main effect of exercise was observed, with lower ileal LPS levels overall in exercised rats than in sedentary rats. Data are presented as mean ± SEM. Statistical analyses were performed using two-way ANOVA with CUMS exposure and exercise as between-subject factors, followed by simple-effects analyses when significant interactions were detected. ^**^P < 0.01, ^***^P < 0.001 for indicated comparisons. TLR4, Toll-like receptor 4; Rela, gene encoding the NF-κB p65/RelA subunit; IL-1β, interleukin-1β; LPS, lipopolysaccharide; HIIT, high-intensity interval training.

For ileal LPS levels, there was a significant main effect of exercise (*F*(1, 35) = 10.306, *P* = 0.003, ηp² = 0.227), whereas neither the main effect of CUMS exposure (*F*(1, 35) = 1.349, *P* = 0.253, ηp² = 0.037) nor the CUMS × exercise interaction (*F*(1, 35) = 0.282, *P* = 0.599, ηp² = 0.008) was significant. Overall, ileal LPS levels were lower in exercised rats than in sedentary rats (**Figure 6B**).

For prefrontal cortical *TLR4* mRNA expression, there was a significant main effect of exercise (*F*(1, 35) = 8.912, *P* = 0.005, ηp^2^ = 0.203), whereas the main effect of CUMS exposure was not significant (*F*(1, 35) = 4.011, *P* = 0.053, ηp^2^ = 0.103). A significant CUMS × exercise interaction was observed (*F*(1, 35) = 26.024, *P* < 0.001, ηp^2^ = 0.426). Simple-effects analyses of the CUMS × exercise interaction showed that, under sedentary conditions, prefrontal cortical *TLR4* mRNA expression was significantly higher in CUMS rats than in control rats (*P* < 0.001), whereas under exercise conditions, *TLR4* mRNA expression was significantly lower in CUMS rats than in control rats (*P* = 0.038). Within CUMS rats, exercise was associated with significantly lower *TLR4* mRNA expression compared with sedentary conditions (*P* < 0.001), whereas no significant exercise-related difference was observed in control rats (*P* = 0.138; **Figure 6C**).

For prefrontal cortical *Rela* mRNA expression, there were significant main effects of CUMS exposure (*F*(1, 35) = 7.695, *P* = 0.009, ηp^2^ = 0.180) and exercise (*F*(1, 35) = 26.760, *P* < 0.001, ηp^2^ = 0.433), as well as a significant CUMS × exercise interaction (*F*(1, 35) = 18.261, *P* < 0.001, ηp^2^ = 0.343). Simple-effects analyses of the CUMS × exercise interaction showed that, under sedentary conditions, prefrontal cortical *Rela* mRNA expression was significantly higher in CUMS rats than in control rats (*P* < 0.001), whereas no significant difference between CUMS and control rats was observed under exercise conditions (*P* = 0.303). Within CUMS rats, exercise was associated with significantly lower *Rela* mRNA expression compared with sedentary conditions (*P* < 0.001), whereas no significant exercise-related difference was observed in control rats (*P* = 0.523; **Figure 6D**).

For prefrontal cortical IL-1β levels, there was a significant main effect of CUMS exposure (*F*(1, 35) = 20.355, *P* < 0.001, ηp^2^ = 0.368), whereas the main effect of exercise was not significant (*F*(1, 35) = 3.222, *P* = 0.081, ηp^2^ = 0.084). A significant CUMS × exercise interaction was observed (*F*(1, 35) = 113.335, *P* < 0.001, ηp^2^ = 0.764). Simple-effects analyses of the CUMS × exercise interaction showed that, under sedentary conditions, prefrontal cortical IL-1β levels were significantly higher in CUMS rats than in control rats (*P* < 0.001), whereas under exercise conditions, IL-1β levels were significantly lower in CUMS rats than in control rats (*P* < 0.001). Within CUMS rats, exercise was associated with significantly lower IL-1β levels compared with sedentary conditions (*P* < 0.001), whereas in control rats, exercise was associated with significantly higher IL-1β levels (*P* < 0.001; **Figure 6E**). Detailed simple-effects estimates and 95% confidence intervals for outcomes showing significant CUMS × exercise interactions are provided in **Supplementary Table 3**.

## 4 Discussion

In the present study, we found that HIIT partially attenuated CUMS-induced depressive-like behaviors and was accompanied by differences in gut microbiota composition identified in exploratory analyses, together with reduced prefrontal cortical LPS levels and lower *TLR4* and *Rela* mRNA expression in the prefrontal cortex. These findings suggest that exercise-induced antidepressant-like effects may be associated with coordinated changes across multiple physiological systems. Consistent with prior work linking depression to dysregulation of the gut-brain-immune axis (Chang et al., 2022; O’Riordan et al., 2025), we observed differences in gut microbiota composition alongside prefrontal cortical LPS-associated inflammatory differences. However, the present study does not establish a direct relationship between these findings. In the following sections, we discuss (1) the behavioral effects of HIIT in the CUMS model and (2) the potential relevance of gut microbiota differences and LPS-associated inflammatory measures, including LPS and IL-1β levels and *TLR4* and *Rela* mRNA expression in the prefrontal cortex.

### 4.1 Changes in rats after CUMS exposure

In this study, we used CUMS as a classic animal model of depression. This model recapitulates several key behavioral and pathophysiological features of depression and reliably induces depressive-like behavior with well-established construct and predictive validity (Willner, 1997; Sharma et al., 2024). Consistent with previous reports (Lin et al., 2005; Chu et al., 2021), after CUMS exposure, rats exhibited markedly attenuated body-weight gain, reduced sucrose preference and rearing frequency, fewer grid crossings, decreased time spent in the center zone, and increased time spent in the corner zone. The reduction in grid crossings indicates lower horizontal locomotor activity in CUMS rats. Accordingly, changes in center- and corner-zone occupancy should be interpreted together with overall locomotor activity rather than as specific measures of anxiety-like behavior in isolation. Because all animals were maintained under a fixed feeding regimen and CUMS rats were additionally exposed to intermittent 24-h food deprivation, the attenuated body-weight gain may reflect the combined physiological and metabolic burden of the overall experimental conditions rather than a stress-specific effect. Importantly, behavioral testing was not performed immediately after the final CUMS stressor. Each OFT assessment during the CUMS modeling period was conducted at least 16 h after completion of the preceding CUMS stressor, and the corresponding SPT was performed on the following day. Therefore, the reduced sucrose preference is unlikely to reflect solely an immediate response to acute food deprivation, although residual or cumulative effects of feeding status cannot be completely excluded. Collectively, the consistent alterations in sucrose preference and open-field behaviors support the development of a depressive-like behavioral phenotype following CUMS exposure.

### 4.2 Changes in rats after HIIT intervention

#### 4.2.1 Effects of HIIT intervention on depressive-like behaviors

The present findings indicate that HIIT produced selective, rather than uniform, improvements in CUMS-associated behavioral abnormalities. The clearest behavioral effect of HIIT was observed for sucrose preference. CUMS_Ex rats showed higher sucrose preference than CUMS_Sed rats at the later assessments, whereas no significant difference was detected at the first assessment during HIIT, suggesting that the improvement in anhedonia-like behavior became detectable with continued training. This finding is broadly consistent with previous studies reporting improved sucrose preference following exercise training in CUMS animals, including studies using aerobic exercise and HIIT (Cai et al., 2023; Wang et al., 2023).

However, the OFT findings showed a more restricted behavioral response. HIIT was associated with higher grid-crossing activity in CUMS rats, whereas no significant exercise-related effects were observed for rearing frequency, center-zone duration, or corner-zone duration. The OFT reflects related but non-equivalent aspects of locomotor, exploratory, and emotional behavior, and locomotor activity can influence the interpretation of zone-occupancy measures (Zhang et al., 2023; Çevik et al., 2025). Accordingly, the increase in grid crossings primarily supports an increase in horizontal locomotor activity, whereas the absence of corresponding changes in rearing or zone occupancy suggests that the behavioral effect did not extend consistently to other exploratory or zone-occupancy measures. Taken together with the SPT findings, these results indicate that the behavioral effects of HIIT in CUMS rats were outcome-specific, with clearer evidence for improvement in anhedonia-like behavior and horizontal locomotor activity. This pattern is broadly consistent with previous experimental studies showing that HIIT can alleviate selected depressive-like behaviors in CUMS animals (Cai et al., 2023; Li et al., 2025).

Body-weight changes provide an additional physiological context for these behavioral findings. Exercised rats showed lower body weight at the later assessments, which may reflect an altered energy balance because the fixed daily food allotment was not increased to compensate for the additional energetic demands of training. However, energy expenditure, body composition, and metabolic status were not directly measured, so the basis of the lower body weight cannot be determined from the present data. Importantly, CUMS_Ex and Con_Ex rats did not differ in Vmax either before HIIT or after 2 weeks of training, while Vmax increased significantly over time. These results indicate that the lower body weight observed during the later phase of training was not accompanied by impaired maximal running performance and are consistent with adaptation to the exercise stimulus. One rat in the CUMS_Ex group died during week 3 of HIIT. Because the cause of death could not be established, its relationship to the intervention remains uncertain and no firm conclusion regarding HIIT tolerability can be drawn from this isolated event.

#### 4.2.2 HIIT-associated differences in gut microbiota diversity and community structure

Previous studies have reported heterogeneous changes in gut microbial alpha diversity in chronic stress models and following exercise interventions (Li et al., 2021; Sheng et al., 2021). In the present study, among the four alpha-diversity indices examined, only the ACE index showed significant effects. ACE values were lower in CUMS_Sed rats than in Con_Sed rats and higher in CUMS_Ex rats than in CUMS_Sed rats, whereas no significant difference was observed between Con_Ex and Con_Sed rats. In contrast, no significant main effects or interactions were detected for the Chao1, Shannon, or Simpson indices. Thus, the present findings provide limited evidence of group differences in microbial richness as reflected by the ACE index, rather than evidence of a generalized alteration in alpha diversity. Given the limited biological replication, these findings should be interpreted cautiously.

With respect to beta diversity, weighted UniFrac-based NMDS showed visual separation among the four experimental groups. The global ANOSIM further indicated that between-group differences in microbial community structure were greater than within-group differences, while the low NMDS stress value indicated that the two-dimensional ordination adequately represented the underlying distance relationships. Previous studies have similarly reported differences in gut microbial community structure between CUMS and control animals, while the specific patterns have varied across stress models and experimental conditions (Sun et al., 2019). These findings are consistent with the possibility that chronic stress is associated with differences in overall gut microbial community structure. Exercise has also been associated with differences in gut microbial community structure, but the direction and magnitude of these responses vary across exercise modalities, intervention durations, host characteristics, and experimental designs (Mika et al., 2015; Mohr et al., 2025). This heterogeneity provides biological context for the overall group-related differences observed in the present study.

Notably, dietary conditions represent an important contextual factor when interpreting the present microbiota findings. Although all groups received the same fixed daily food allotment, thereby standardizing food availability across groups, the feeding regimen itself may have influenced gut microbial composition and metabolism and contributed to the endpoint microbial profiles observed in this study (Kern et al., 2023; Wang et al., 2024).

#### 4.2.3 HIIT-associated differences in gut microbiota taxonomic composition

To further characterize group-related differences in gut microbiota composition, we examined taxonomic profiles at the phylum and genus levels. Bacillota and Bacteroidota were the predominant phyla across the four groups, which is broadly consistent with the dominant gut microbial composition reported in rodent studies of chronic stress and depression (Wei et al., 2019; Liu et al., 2021). However, previous studies have also shown substantial heterogeneity in the specific taxonomic alterations associated with chronic stress. A recent review comparing gut microbiota across animal models of stress and depression found consistent evidence of stress-associated alterations in microbial composition, while the specific taxa involved and the patterns of change varied across studies and models (Herselman et al., 2022). Given the limited biological replication in the present microbiota analysis, the taxon-level findings represent exploratory group differences that warrant further validation in larger studies.

At the phylum level, the adjusted pairwise analysis showed that Pseudomonadota was more abundant in CUMS_Sed rats than in Con_Sed rats. Similar increases in Proteobacteria, currently classified as Pseudomonadota, have also been reported in previous CUMS studies (Cai et al., 2022; Chen et al., 2024). In contrast, other CUMS studies have reported reductions in Proteobacteria (Tan et al., 2022; Gao et al., 2024). Such inconsistency may reflect differences in animal strain, stress paradigm, diet, sampling procedures, sequencing methods, and statistical approaches. These divergent findings indicate that changes in Pseudomonadota are not uniform across chronic stress models and should therefore be interpreted within the specific experimental context.

In addition to the CUMS-associated phylum-level differences, exercise-associated phylum-level differences were observed in both non-stressed and CUMS conditions. Under non-stressed conditions, Con_Ex rats showed a lower relative abundance of Bacillota but higher relative abundances of Bacteroidota and Spirochaetota than Con_Sed rats. In contrast, under CUMS conditions, CUMS_Ex rats showed a lower relative abundance of Cyanobacteriota than CUMS_Sed rats. Because formal stress × exercise interactions were not tested at the taxon level, these results provide a descriptive comparison of exercise-associated taxonomic differences under the two conditions. Previous studies have likewise reported substantial heterogeneity in exercise-associated gut microbial profiles, with variation related to exercise modality, intervention duration, host characteristics, and experimental design (Cullen et al., 2023).

At the genus level, the adjusted pairwise analyses further indicated that CUMS-associated differences and exercise were characterized by distinct sets of taxa. Under sedentary conditions, CUMS_Sed rats had lower relative abundances of *Muribaculum*, *Parasutterella*, Coriobacteriaceae UCG-002, UG within Atopobiaceae, and *Dubosiella*, but higher relative abundances of *Odoribacter*, UG within Prevotellaceae, UT within Bacteroidales, *Bilophila*, and *Parabacteroides* than Con_Sed rats. Several of these taxa have metabolic or immunological properties that may be relevant to stress-associated host physiology. However, because much of the available functional evidence is species- or strain-specific, the following studies are used primarily to provide biological context for the genus-level differences observed here, while their specific functional relevance requires further validation.

*Muribaculum* and related members of Muribaculaceae are involved in the utilization of complex carbohydrates and have been associated with short-chain fatty acid production. Recent experimental evidence further showed that supplementation with *Muribaculum intestinale* increased short-chain fatty acids and attenuated depressive-like behaviors in stressed mice (Pérez Escriva et al., 2022; Zhu et al., 2024; He et al., 2026). Similarly, *Parasutterella* has been characterized as a succinate-producing commensal that can influence bile acid and aromatic amino acid metabolism, and recent studies have implicated *Parasutterella*-associated bile acid metabolism in stress-related behavioral phenotypes (Ju et al., 2019; Zhou et al., 2023). Conversely, the higher relative abundance of *Bilophila* in CUMS_Sed rats is noteworthy because *Bilophila wadsworthia* is a Gram-negative anaerobe involved in sulfur metabolism and has been associated with intestinal inflammatory responses (Liu et al., 2023; Pimenta et al., 2024; Yoshida et al., 2025). However, the present 16S data do not establish species identity or demonstrate altered microbial metabolite production or inflammatory activity. Together, these observations highlight the diverse biological characteristics reported for the taxa that differed between CUMS_Sed and Con_Sed rats, while their metabolic and immunological relevance in the present study remains uncertain.

*Parabacteroides* is particularly noteworthy because its relative abundance was higher in CUMS_Sed rats than in Con_Sed rats and lower in CUMS_Ex rats than in CUMS_Sed rats. This pattern indicates opposite group-associated differences in Parabacteroides relative abundance under CUMS and exercise conditions. Previous studies have reported both potentially adverse and beneficial associations of *Parabacteroides* with depression-related phenotypes, suggesting that its biological relevance may depend on species or strain identity, host physiological state, microbial community context, and associated metabolic functions (Gomez-Nguyen et al., 2021; Yang et al., 2021; Liang et al., 2022; Ma et al., 2022; Cheng et al., 2024). Accordingly, the lower relative abundance of Parabacteroides in CUMS_Ex rats should be interpreted as an exercise-associated taxonomic pattern rather than evidence that this genus contributed to the behavioral effects of HIIT.

The genus-level taxa associated with exercise also differed between the non-stressed and CUMS comparisons. Under non-stressed conditions, Con_Ex rats had higher relative abundances of Rikenellaceae RC9 gut group, Prevotellaceae UCG-001, *Quinella*, UG within Spirochaetaceae, and *Bacteroides*, together with a lower relative abundance of *Clostridium sensu stricto 1*, than Con_Sed rats. These taxa have been variably associated with exercise and host metabolic phenotypes in previous studies, but their functional relevance remains incompletely characterized (Song et al., 2019; Rebelo et al., 2022; Salvador et al., 2023; Watanabe et al., 2023; Bellar et al., 2026).

Under CUMS conditions, *Peptococcus*, UT within Gastranaerophilales, *Parabacteroides*, and *Paenalcaligenes* were all less abundant in CUMS_Ex rats than in CUMS_Sed rats. Because the functional relevance of these taxa may depend on species- or strain-level characteristics, their biological significance in the present study remains uncertain. Thus, the present findings primarily indicate HIIT-associated differences in the relative abundance of these taxa and do not provide sufficient evidence for specific functional inferences.

Taken together, the exercise-associated taxa showed limited overlap between the non-stressed and CUMS comparisons, suggesting that the taxonomic patterns accompanying exercise may differ across physiological contexts. However, because these observations were based on pairwise comparisons rather than formal taxon-specific interaction tests, they should not be interpreted as evidence that CUMS modified the microbial response to exercise. Notably, the exercise-associated taxonomic profile in CUMS rats did not simply converge toward that observed in sedentary controls. Rather, the present findings suggest that exercise was associated with distinct endpoint taxonomic profiles under non-stressed and CUMS conditions. Given the limited biological replication and terminal-only microbiota sampling, the present study could not assess longitudinal microbiota changes during CUMS exposure or HIIT intervention, nor were individual-level associations between microbial features and behavioral or inflammatory outcomes examined. Thus, the observed taxonomic differences primarily represent exploratory group-level patterns, and their relationships with host phenotypes and specific microbial mechanisms require further investigation.

#### 4.2.4 Effects of HIIT on LPS-associated inflammatory measures and *TLR4/Rela* mRNA expression in CUMS rats

In the present study, compared with the Con_Sed group, the CUMS_Sed group exhibited higher LPS levels in the prefrontal cortex, accompanied by higher *TLR4* and *Rela* mRNA expression and increased IL-1β levels. These findings suggest that CUMS was associated with prefrontal cortical inflammatory alterations with endotoxin-related features and may reflect enhanced LPS-associated inflammatory responses in the prefrontal cortex. Such alterations may be related to a proinflammatory state associated with depressive-like phenotypes (Cheng et al., 2016; Wang et al., 2018). However, because only *TLR4* and *Rela* mRNA expression was assessed, the present data do not provide direct evidence of TLR4/NF-κB pathway activation. Such activation would require confirmation using protein-level or subcellular markers, such as IκB degradation, p65 phosphorylation, or nuclear translocation.

Consistent with prior studies showing that exercise training can influence inflammation-related measures (Qu et al., 2020), HIIT was associated with lower prefrontal cortical LPS levels, *TLR4* and *Rela* mRNA expression, and IL-1β levels in CUMS rats. Although *TLR4* mRNA expression in the CUMS_Ex group was numerically lower than that in the Con_Sed group, this difference was not statistically significant (P = 0.498) and should not be interpreted as suppression below the non-stressed sedentary level. Importantly, the inflammatory response to HIIT differed according to stress status. In control rats, IL-1β levels were significantly higher in the Con_Ex group than in the Con_Sed group, whereas LPS, *TLR4*, and *Rela* did not differ significantly between the two control groups. Thus, the present findings suggest that HIIT may attenuate stress-associated prefrontal inflammatory alterations in CUMS rats, but do not support a uniform anti-inflammatory effect across physiological conditions.

Notably, ileal LPS levels showed a significant main effect of exercise without a CUMS × exercise interaction, whereas prefrontal cortical LPS showed a significant CUMS × exercise interaction, with lower levels in CUMS_Ex rats than in CUMS_Sed rats. These tissue-specific patterns do not support a simple correspondence between ileal and prefrontal cortical LPS levels. Moreover, ileal tissue LPS represents LPS present within the sampled intestinal tissue and does not directly indicate intestinal permeability, systemic endotoxin exposure, or the extent of gut-derived LPS translocation.

Previous studies nevertheless provide several biologically plausible pathways through which peripheral endotoxin-related processes may influence central inflammation. Increased intestinal permeability can facilitate the entry of microbial products, including LPS, into the circulation, while systemic LPS can promote inflammatory signaling and affect blood-brain barrier integrity and neuroimmune responses. Exercise may also influence intestinal barrier function, but these effects appear to depend on exercise intensity, duration, and physiological context. Regular moderate exercise has frequently been associated with preservation of intestinal barrier integrity, whereas high-intensity exercise can transiently increase intestinal permeability and tight-junction disruption (Dmytriv et al., 2024; Takami et al., 2024). These observations provide a possible biological framework for considering peripheral and central LPS-associated responses, but they do not establish the pathway responsible for the findings observed in the present study.

Because intestinal permeability, circulating LPS, LPS translocation and clearance, and BBB function were not directly assessed, the source of the prefrontal cortical LPS signal and its relationship to intestinal LPS cannot be determined. In particular, the taxonomic differences identified in the exploratory microbiota analysis should not be used to infer changes in total gut-derived LPS production or exposure. Future studies combining intestinal barrier markers, circulating endotoxin-related measures, and central inflammatory assessments will be required to determine whether peripheral LPS-related processes contribute to the prefrontal cortical responses associated with HIIT.

### 4.3 Limitations

This study has several limitations. First, the inflammatory assessment focused primarily on LPS-associated measures involving *TLR4* and *Rela* in the prefrontal cortex. Other inflammatory and non-inflammatory pathways, as well as other brain regions involved in stress and emotion regulation, such as the hippocampus and amygdala, were not examined. In addition, TLR4/NF-κB-related inflammatory processes were evaluated using LPS levels, *TLR4* and *Rela* mRNA expression, and IL-1β levels, whereas protein-level indicators of NF-κB activation were not assessed. Therefore, the present findings do not provide direct evidence of activation or inhibition of the TLR4/NF-κB signaling pathway. Second, the gut microbiota analysis included only three biological samples per group, which substantially limits the statistical power, robustness, and generalizability of the microbiota findings. Microbiota samples were collected only at the terminal time point, and no post-CUMS/pre-HIIT samples were available, precluding assessment of longitudinal within-animal microbiota changes. In addition, all animals underwent a 12-h food and water deprivation period before terminal sampling, which may have influenced the observed microbial profiles. Individual-level associations between microbiota features and behavioral or inflammatory outcomes were not assessed because of the limited microbiota sample size. Third, intestinal barrier integrity, circulating LPS, systemic inflammatory markers, LPS translocation and clearance, and blood-brain barrier function were not directly assessed. Therefore, the present study cannot determine whether gut-derived LPS contributed to the prefrontal cortical findings or establish a causal gut-to-brain LPS pathway. Moreover, although LPS measurements were standardized within each tissue type using the same wet tissue mass across samples and a fixed tissue-to-buffer ratio, assay performance in brain homogenate was not independently validated using matrix-specific spike-recovery or dilution-parallelism experiments, and samples were not normalized to total protein concentration. Accordingly, prefrontal cortical LPS values should be interpreted cautiously and primarily as within-tissue group differences. Fourth, although exercise intensity was individualized according to Vmax and Vmax increased after 2 weeks of training, internal physiological load was not directly assessed using measures such as oxygen consumption, blood lactate, or energy expenditure. Therefore, equivalent metabolic or cardiorespiratory workload between groups cannot be established. Fifth, all animals received the same fixed daily food allotment, whereas CUMS rats were additionally exposed to intermittent food deprivation as part of the stress paradigm. Therefore, residual or cumulative effects of feeding status on body-weight gain, sucrose-related motivation, and gut microbiota cannot be excluded. Because no ad libitum-fed control group was included, the independent contribution of the fixed feeding regimen and its potential interaction with the experimental conditions cannot be determined. Sixth, repeated OFT exposure may have produced between-session habituation. Although all groups followed the same testing schedule and test-related effects were included in the statistical models, habituation may have contributed to longitudinal changes in exploratory and locomotor behavior. Finally, only male rats were included in the present study, which limits the generalizability of the findings across sexes. Future studies should determine whether the behavioral, microbial, and inflammatory responses to HIIT are also observed in female animals.

## 5 Conclusion

CUMS rats showed marked depressive-like behaviors accompanied by differences in gut microbiota composition and increased prefrontal cortical LPS-associated inflammatory measures. HIIT partially improved depressive-like behaviors and was accompanied by differences in gut microbiota composition, lower prefrontal cortical LPS and IL-1β levels, and lower *TLR4* and *Rela* mRNA expression in CUMS rats. These findings suggest that the behavioral effects of HIIT were associated with differences in gut microbiota composition and prefrontal cortical LPS-associated inflammatory measures. Causal relationships between these biological differences and the behavioral effects of HIIT remain to be established.

## Data Availability

The original contributions presented in the study are included in the article/**Supplementary Material**. Further inquiries can be directed to the corresponding author. The 16S rRNA gene sequencing data generated in this study have been deposited in the NCBI Sequence Read Archive (SRA) under BioProject accession number PRJNA1535158 (https://www.ncbi.nlm.nih.gov/bioproject/PRJNA1535158).
