# Supplementary material for: High-intensity interval training alleviates depressive-like behaviors and is associated with differences in gut microbiota composition and prefrontal LPS-associated inflammatory measures in CUMS rats
Source: Front Cell Infect Microbiol. 2026 Oct 1;16:1929000. doi: 10.3389/fcimb.2026.1929000 (PMC13668571; doi:10.3389/fcimb.2026.1929000)
Supplement: Supplementary file 1 [file Table1.docx]

**Supplementary Table 1. Simple-effects comparisons for body weight and behavioral outcomes before the HIIT intervention**

| **Outcome** | **Assessment** | **Comparison** | **Mean difference** | **SE** | **95% CI** | ***P*** |
| --- | --- | --- | --- | --- | --- | --- |
| Body weight (g) | Week 0 | Con - CUMS | 2.991 | 2.128 | -1.317 to 7.298 | 0.168 |
|  | Week 1 | Con - CUMS | 38.431 | 3.410 | 31.528 to 45.333 | < 0.001 |
|  | Week 2 | Con - CUMS | 57.611 | 4.177 | 49.155 to 66.066 | < 0.001 |
|  | Week 3 | Con - CUMS | 58.710 | 3.812 | 50.993 to 66.428 | < 0.001 |
|  | Week 4 | Con - CUMS | 66.838 | 5.014 | 56.688 to 76.989 | < 0.001 |
| Corner-zone duration (s) | Test 1 | Con - CUMS | -43.069 | 7.597 | -58.448 to -27.689 | < 0.001 |
|  | Test 2 | Con - CUMS | -71.200 | 6.230 | -83.811 to -58.589 | < 0.001 |
|  | Test 2 vs. Test 1 | Con: Test 2 - Test 1 | 3.150 | 7.095 | -11.214 to 17.514 | 0.660 |
|  |  | CUMS: Test 2 - Test 1 | 31.282 | 7.095 | 16.917 to 45.646 | < 0.001 |

Note: For between-group comparisons, mean differences were calculated as Con minus CUMS. For within-group time comparisons, mean differences were calculated as Test 2 minus Test 1. Simple-effects comparisons were based on estimated marginal means with Fisher’s least significant difference (LSD) adjustment. Simple-effects comparisons are presented for outcomes showing significant interactions in the main analyses. CI, confidence interval; SE, standard error; CUMS, chronic unpredictable mild stress; Con, control.
