# Supplementary material for: High-intensity interval training alleviates depressive-like behaviors and is associated with differences in gut microbiota composition and prefrontal LPS-associated inflammatory measures in CUMS rats
Source: Front Cell Infect Microbiol. 2026 Oct 1;16:1929000. doi: 10.3389/fcimb.2026.1929000 (PMC13668571; doi:10.3389/fcimb.2026.1929000)
Supplement: Supplementary file 2 [file Table2.docx]

**Supplementary Table 2. Simple-effects comparisons for body weight and behavioral outcomes during and after the HIIT intervention**

| **Outcome** | **Assessment** | **Comparison** | **Mean difference** | **SE** | **95% CI** | ***P*** |
| --- | --- | --- | --- | --- | --- | --- |
| **Body weight (g)** | Week 5 | Con - CUMS, averaged across exercise | 42.604 | 5.248 | 31.951 to 53.257 | < 0.001 |
|  | Week 6 |  | 32.416 | 5.439 | 21.374 to 43.458 | < 0.001 |
|  | Week 7 |  | 26.982 | 5.671 | 15.470 to 38.494 | < 0.001 |
|  | Week 8 |  | 26.390 | 5.621 | 14.979 to 37.801 | < 0.001 |
|  | Week 9 |  | 20.647 | 6.060 | 8.344 to 32.949 | 0.002 |
|  | Week 5 | Ex - Sed, averaged across CUMS | 5.086 | 5.248 | -5.567 to 15.739 | 0.339 |
|  | Week 6 |  | 1.834 | 5.439 | -9.208 to 12.876 | 0.738 |
|  | Week 7 |  | -3.032 | 5.671 | -14.544 to 8.480 | 0.596 |
|  | Week 8 |  | -15.740 | 5.621 | -27.151 to -4.329 | 0.008 |
|  | Week 9 |  | -16.817 | 6.060 | -29.119 to -4.514 | 0.009 |
| **Sucrose preference (%)** | Test 3 | CUMS: Ex - Sed | -3.078 | 4.112 | -11.425 to 5.270 | 0.459 |
|  | Test 4 |  | 8.789 | 3.801 | 1.072 to 16.506 | 0.027 |
|  | Test 5 |  | 7.556 | 2.737 | 1.998 to 13.113 | 0.009 |
|  | Test 3 | Con: Ex - Sed | 4.500 | 4.002 | -3.625 to 12.625 | 0.268 |
|  | Test 4 |  | 2.900 | 3.700 | -4.611 to 10.411 | 0.438 |
|  | Test 5 |  | 0.700 | 2.664 | -4.709 to 6.109 | 0.794 |
| **Center-zone duration (s)** | Test 3 | Con - CUMS, averaged across exercise | 17.761 | 3.812 | 10.022 to 25.501 | < 0.001 |
|  | Test 4 |  | 19.949 | 2.749 | 14.368 to 25.531 | < 0.001 |
|  | Test 5 |  | 17.550 | 3.100 | 11.257 to 23.842 | < 0.001 |
|  | Test 3 | Ex - Sed, averaged across CUMS | -1.562 | 3.812 | -9.302 to 6.177 | 0.684 |
|  | Test 4 |  | 2.352 | 2.749 | -3.230 to 7.933 | 0.398 |
|  | Test 5 |  | 4.949 | 3.100 | -1.343 to 11.242 | 0.119 |
| **Grid crossings (times)** | Tests 3-5 averaged | Con: Ex - Sed | 1.900 | 2.991 | -4.172 to 7.972 | 0.529 |
|  | Tests 3-5 averaged | CUMS: Ex - Sed | 27.330 | 3.073 | 21.091 to 33.568 | < 0.001 |

Note: Comparisons were based on estimated marginal means from three-way mixed-design repeated-measures ANOVAs. Simple-effects comparisons for significant two-way interactions were averaged across the third factor. SPT comparisons were performed within each CUMS condition at each assessment following the significant three-way interaction. Fisher’s LSD adjustment was used. Complete cases were analyzed (Con_Sed, n = 10; Con_Ex, n = 10; CUMS_Sed, n = 10; CUMS_Ex, n = 9). CI, confidence interval; SE, standard error; SPT, sucrose preference test; CUMS, chronic unpredictable mild stress; Con, control; Sed, sedentary; Ex, exercise.
