# Supplementary material for: High-intensity interval training alleviates depressive-like behaviors and is associated with differences in gut microbiota composition and prefrontal LPS-associated inflammatory measures in CUMS rats
Source: Front Cell Infect Microbiol. 2026 Oct 1;16:1929000. doi: 10.3389/fcimb.2026.1929000 (PMC13668571; doi:10.3389/fcimb.2026.1929000)
Supplement: Supplementary file 3 [file Table3.docx]

**Supplementary Table 3. Simple-effects comparisons for biochemical outcomes showing significant CUMS × exercise interactions**

| **Outcome** | **Comparison** | **Mean difference** | **SE** | **95% CI** | ***P*** |
| --- | --- | --- | --- | --- | --- |
| **Prefrontal cortical LPS (EU/L)** | CUMS_Sed - Con_Sed | 283.900 | 51.904 | 178.529 to 389.271 | < 0.001 |
|  | CUMS_Ex - Con_Ex | 104.760 | 53.326 | -3.498 to 213.018 | 0.057 |
|  | Con_Ex - Con_Sed | -8.030 | 51.904 | -113.401 to 97.341 | 0.878 |
|  | CUMS_Ex - CUMS_Sed | -187.170 | 53.326 | -295.428 to -78.912 | 0.001 |
| **Prefrontal cortical TLR4 mRNA** | CUMS_Sed - Con_Sed | 0.940 | 0.185 | 0.565 to 1.315 | < 0.001 |
|  | CUMS_Ex - Con_Ex | -0.410 | 0.190 | -0.795 to -0.025 | 0.038 |
|  | Con_Ex - Con_Sed | 0.280 | 0.185 | -0.095 to 0.655 | 0.138 |
|  | CUMS_Ex - CUMS_Sed | -1.070 | 0.190 | -1.455 to -0.685 | < 0.001 |
| **Prefrontal cortical Rela mRNA** | CUMS_Sed - Con_Sed | 0.470 | 0.093 | 0.281 to 0.659 | < 0.001 |
|  | CUMS_Ex - Con_Ex | -0.100 | 0.096 | -0.294 to 0.094 | 0.303 |
|  | Con_Ex - Con_Sed | -0.060 | 0.093 | -0.249 to 0.129 | 0.523 |
|  | CUMS_Ex - CUMS_Sed | -0.630 | 0.096 | -0.824 to -0.436 | < 0.001 |
| **Prefrontal cortical IL-1β (pg/g tissue)** | CUMS_Sed - Con_Sed | 436.600 | 40.181 | 355.029 to 518.171 | < 0.001 |
|  | CUMS_Ex - Con_Ex | -176.690 | 41.282 | -260.497 to -92.883 | < 0.001 |
|  | Con_Ex - Con_Sed | 358.350 | 40.181 | 276.779 to 439.921 | < 0.001 |
|  | CUMS_Ex - CUMS_Sed | -254.940 | 41.282 | -338.747 to -171.133 | < 0.001 |

Note: Comparisons were based on estimated marginal means from two-way ANOVAs with CUMS exposure and exercise as between-subject factors. Simple-effects analyses were performed for outcomes showing significant CUMS × exercise interactions. Mean differences were calculated as the first group minus the second group, with Fisher’s LSD adjustment. Sample sizes were n = 10 for Con_Sed, Con_Ex, and CUMS_Sed and n = 9 for CUMS_Ex. CI, confidence interval; SE, standard error; LPS, lipopolysaccharide; TLR4, Toll-like receptor 4; Rela, gene encoding the NF-κB p65/RelA; CUMS, chronic unpredictable mild stress; Sed, sedentary; Ex, exercise.
